# Performance of virtual screening against GPCR homology models: Impact of template selection and treatment of binding site plasticity

**DOI:** 10.1371/journal.pcbi.1007680

**Published:** 2020-03-13

**Authors:** Mariama Jaiteh, Ismael Rodríguez-Espigares, Jana Selent, Jens Carlsson

**Affiliations:** 1 Science for Life Laboratory, Department of Cell and Molecular Biology, Uppsala University, Uppsala, Sweden; 2 Research Programme on Biomedical Informatics (GRIB), Department of Experimental and Health Sciences of Pompeu Fabra University (UPF), Hospital del Mar Medical Research Institute (IMIM), Barcelona, Spain; University of Maryland School of Pharmacy, UNITED STATES

## Abstract

Rational drug design for G protein-coupled receptors (GPCRs) is limited by the small number of available atomic resolution structures. We assessed the use of homology modeling to predict the structures of two therapeutically relevant GPCRs and strategies to improve the performance of virtual screening against modeled binding sites. Homology models of the D_2_ dopamine (D_2_R) and serotonin 5-HT_2A_ receptors (5-HT_2A_R) were generated based on crystal structures of 16 different GPCRs. Comparison of the homology models to D_2_R and 5-HT_2A_R crystal structures showed that accurate predictions could be obtained, but not necessarily using the most closely related template. Assessment of virtual screening performance was based on molecular docking of ligands and decoys. The results demonstrated that several templates and multiple models based on each of these must be evaluated to identify the optimal binding site structure. Models based on aminergic GPCRs showed substantial ligand enrichment and there was a trend toward improved virtual screening performance with increasing binding site accuracy. The best models even yielded ligand enrichment comparable to or better than that of the D_2_R and 5-HT_2A_R crystal structures. Methods to consider binding site plasticity were explored to further improve predictions. Molecular docking to ensembles of structures did not outperform the best individual binding site models, but could increase the diversity of hits from virtual screens and be advantageous for GPCR targets with few known ligands. Molecular dynamics refinement resulted in moderate improvements of structural accuracy and the virtual screening performance of snapshots was either comparable to or worse than that of the raw homology models. These results provide guidelines for successful application of structure-based ligand discovery using GPCR homology models.

## Author summary

Three-dimensional structures of proteins combined with computational methods have become widely used to identify starting-points for drug discovery. However, this powerful approach is limited by the lack of atomic resolution structures for many drug targets. G protein-coupled receptors (GPCRs) belong to the largest family of cell surface receptors and play roles in numerous physiological processes. As GPCRs are important therapeutic targets, there is significant interest in applying structure-based *in silico* screening to accelerate the drug discovery process. However, GPCRs have been notoriously difficult to crystallize and structures are lacking for >80% of the family. We assessed prediction of GPCR structure based on previously determined crystal structures as templates by using the homology modeling method. We explored strategies to identify models suitable for virtual screening with the molecular docking method and to further refine structures using molecular dynamics simulations. Our calculations revealed that the closest homologue of a target is not necessarily the best template and demonstrated how accurate binding site models with excellent ability to identify ligands can be obtained. The results highlight strengths and weaknesses of structure prediction methods and provide guidelines for successful application of virtual screening to proteins of unknown structure.

## Introduction

G protein-coupled receptors (GPCRs) constitute a large superfamily of membrane proteins and play key roles in cellular signaling. GPCRs recognize chemically diverse molecules and binding of their endogenous ligands leads to activation of intracellular signaling pathways [1]. As GPCRs control numerous physiological processes, compounds that either stimulate or block their activity are valuable tools to understand receptor function and have therapeutic applications. Efforts to develop drugs that target GPCRs have been remarkably successful. Currently, 34% of medications approved by the US Food and Drug Administration mediate their effects via GPCRs and are used to treat a wide range of conditions, *e*.*g*. cardiovascular, neuropsychiatric, and neurodegenerative diseases [2].

Advances in structural biology during the last decade led to a rapid increase of the number of atomic resolution structures of GPCRs. Structures of 64 unique GPCRs have been solved, providing opportunities to use molecular modeling to accelerate drug discovery. Structure-based virtual screens of large chemical libraries against GPCRs, followed by experimental testing of top-scoring compounds, have successfully identified leads of many therapeutic targets, including biogenic amine [3–8], purine [9], peptide [10,11] and protein-binding receptors [12]. The fact that molecular docking can be used to search chemical libraries with >100 million compounds [8] and identify ligands with hit rates as high as 73% [4] suggests that virtual screening can make important contributions to drug discovery.

Despite the increasing number of experimentally determined GPCR structures, crystallization remains challenging and atomic resolution information is lacking for >80% of the non-olfactory receptors [13]. One approach to circumvent this problem is protein structure prediction using homology modeling. A template with >30% sequence identity to the target is considered sufficient to use homology modeling, but the utility of the resulting structures will depend on the application [14]. As reflected by community-wide GPCR structure prediction assessments, modeling of receptor-drug complexes is challenging. Although templates with >35% sequence identity were available, only a small number of research groups identified ligand binding modes close to the experimentally determined complexes for the A_2A_ adenosine, D_3_ dopamine, 5-HT_1B_ and 5-HT_2B_ serotonin receptors. No research group was able to accurately model ligand binding modes if only distant templates were available, *e*.*g*. for the CXCR4 and smoothened GPCRs [15–17].

Reliable techniques for GPCR modeling would make it possible to extend the use of structure-based ligand discovery to many unexplored drug targets. An increasingly employed strategy to evaluate models is to use molecular docking to assess if the binding site can identify known active compounds among decoys [18–24]. A model that displays high enrichment of known ligands is considered to be a good representative of the receptor structure and suitable for virtual screening. Despite the challenges involved in predicting the structures of GPCR-ligand complexes, several virtual screens against homology models have been successful [12,25–31]. For example, Carlsson *et al*. demonstrated that a high quality model of the D_3_ dopamine receptor performed as well as a crystal structure in prospective molecular docking screens [29]. However, it should be noted that virtual screening based on GPCR homology models has several caveats. First, it is not clear if the approach is restricted to targets for which closely related templates are available. Prediction of drug complexes will be sensitive to conformations of side chains and loop regions forming the binding site, which are difficult to model based on distant templates. A common rule of thumb is to select the template with the highest sequence identity to construct a homology model. However, benchmarks of homology modeling have not found any clear correlation between the sequence identity of the template and virtual screening performance [32] and, intriguingly, GPCR models based on distant templates have occasionally resulted in better ligand enrichment than closely related ones [33,34]. Second, prospective docking screens against homology models are generally carried out against a single rigid structure of the binding site. As GPCRs are flexible proteins, a model with static side chains will not capture induced-fit effects and may fail to identify ligands that are recognized by different conformations. Consideration of multiple receptor conformations has the potential to improve ligand enrichment, but will also increase the computational cost of virtual screening. Finally, a homology model will contain errors originating from structural differences between the target and template, which are likely to increase with decreasing sequence identity. Refinement of homology models with more rigorous methods such as molecular dynamics (MD) simulations could lead to improved structures. However, the accuracy and virtual screening performance of raw and MD-refined homology models have rarely been compared [31,35].

In this work, we explored strategies to identify GPCR homology models suitable for virtual screening. The D_2_ dopamine receptor (D_2_R) and 5-HT_2A_ serotonin receptor (5-HT_2A_R) were selected as targets. The D_2_R and 5-HT_2A_R belong to the family of aminergic receptors and modulation of both targets is essential for the therapeutic effect of many antipsychotic drugs [36]. Homology models based on 16 crystal structure templates, representing both closely and distantly related receptors, were generated. The models were evaluated based on their agreement with recently determined D_2_R and 5-HT_2A_R crystal structures, and sets of known ligands were docked to the binding sites to evaluate their virtual screening performance. We also investigated if predictions could be improved by using ensembles of homology models or by refinement with MD simulations. Finally, we assessed the influence of extracellular loop regions on virtual screening performance and the possibility that the choice of template may inflict bias toward certain ligand chemotypes.

## Results

### Homology modeling based on 16 different templates

Homology models of the D_2_R and 5-HT_2A_R were generated based on crystal structures of 12 aminergic receptors and four non-aminergic GPCRs (Table 1). Representative structures of two adrenergic (β_1_AR [37] and β_2_AR [38]), two dopamine (D_3_R [39] and D_4_R [40]), one histamine (H_1_R [41]), three serotonin (5-HT_1B_R [42], 5-HT_2B_R [43], and 5-HT_2C_R [44]), and four muscarinic (M_1_R [45], M_2_R [46], M_3_R [47], and M_4_R [45]) receptors were selected as templates. A set of more distantly related non-aminergic templates was selected to cover GPCRs that recognize different types of ligands and included the Rhodopsin (Rho [48]), CXCR4 chemokine (CXCR4 [49]), A_2A_ adenosine (A_2A_AR [50]), and Cannabinoid 1 (CB1R [51]) receptors. In order to enable comparisons of the homology models to the experimental D_2_R or 5-HT_2A_R structures, which were crystallized in inactive conformations, templates determined in inactive states were selected in a majority of the cases. In a few instances, templates crystallized in intermediate conformations were used (5-HT_1B_R and 5-HT_2B_R). Sequence alignments were facilitated by the strongly conserved topology of GPCRs and manually adjusted for the second extracellular loop (ECL2) as this region is involved in ligand recognition (S1 and S2 Files). The 16 templates covered a wide range of transmembrane helix (TM) and binding site (BS) sequence identities with the D_2_R and 5-HT_2A_R (Table 1), ranging from 21% to 77% and 6% to 94%, respectively. Crystal structures of the 5-HT_2C_R and D_3_R shared the highest TM sequence identities with the 5-HT_2A_R (75%) and D_2_R (77%), respectively. As expected, the TM and BS sequence identities to the target receptors were low for the four non-aminergic templates (21–37% and 6–19%, respectively).

**Table 1:** Crystal structures selected as templates for homology modeling of the D_2_R and 5-HT_2A_R.

Receptors ^a^	PDB code^b^	Sequence identity (%)			
		D_2_R		5HT_2A_R	
		TM^c^	BS^c^	TM^c^	BS^c^
β_1_ Adrenergic (β_1_AR)	2VT4	44	59	41	59
β_2_ Adrenergic (β_2_AR)	2RH1	41	56	39	63
D_3_ Dopamine (D_3_R)	3PBL	77	94	41	53
D_4_ Dopamine (D_4_R)	5WIU	51	71	39	50
H_1_ Histamine (H_1_R)	3RZE	38	34	32	41
5-HT_1B_ (5-HT_1B_R)	4IAR	46	47	36	44
5-HT_2B_ (5-HT_2B_R)	4IB4	40	47	66	75
5-HT_2C_ (5-HT_2C_R)	6BQH	45	53	75	88
M_1_ Muscarinic (M_1_R)	5CXV	35	19	31	25
M_2_ Muscarinic (M_2_R)	3UON	34	22	29	25
M_3_ Muscarinic (M_3_R)	4DAJ	36	22	35	31
M_4_ Muscarinic (M_4_R)	5DSG	36	25	30	28
Rhodopsin (Rho)	1F88	25	19	21	19
CXCR4 chemokine (CXCR4)	3ODU	25	13	22	9
A_2A_ Adenosine (A_2A_AR)	4EIY	37	13	30	16
Cannabinoid 1 (CB1R)	5U09	26	6	22	9

^a^ Receptor names and abbreviations.

^b^ PDB code of the crystal structure used as template.

^c^ The TM sequence identity was calculated based on the residues specified in S1 Table.

^d^ The BS was defined using the definition of Michino *et al*.[52] and only included residues in the TM region.

For each template, the program MODELLER [53] was used to generate a set of 250 homology models per target, from which the 50 structures with the best DOPE scores [54] were extracted for analyses. Homology models derived from the same template varied in the side chain conformations and in the backbone of the loops due to gaps or insertions in the alignment. For models based on the same template, the average pairwise RMSDs of side chains in the binding site ranged from 0.9 to 2.0 Å (S2 Table). The largest variation in binding site structure was obtained for the non-aminergic templates whereas the lowest was obtained from the template with the highest BS sequence identity. Models obtained from different templates also displayed variation in the backbone structure due to differences in the relative orientations of the helices and loop regions.

### Comparison of homology models to crystal structures

**Fig 1:**
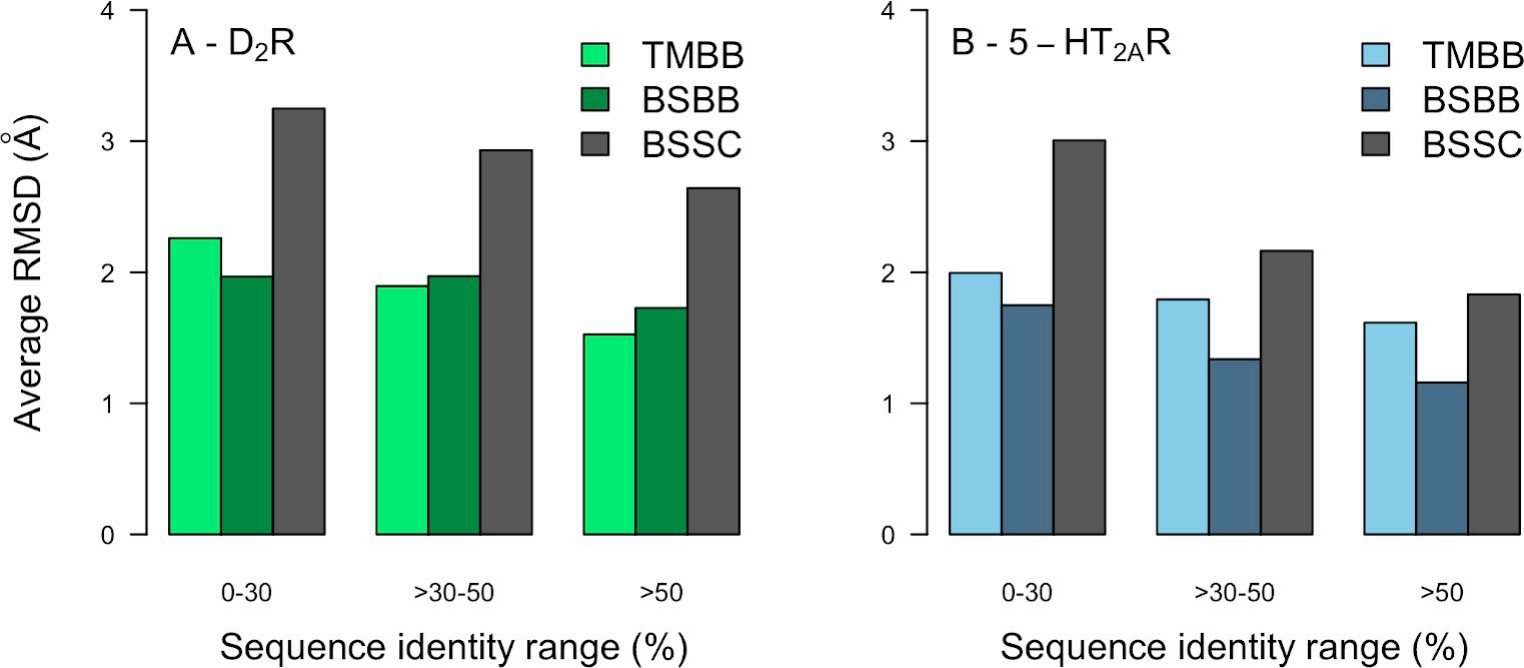
Structural accuracy of homology models based on templates with different levels of sequence identity. The average RMSD_TMBB_ (green and blue bars), RMSD_BSBB_ (dark green and blue bars), and RMSD_BSSC_ (grey bars) to the crystal structures were calculated for 50 models per template. Bar charts of structural accuracy using templates with different levels of sequence identity was calculated for (A) the D_2_R and (B) 5-HT_2A_R.

**Fig 2:**
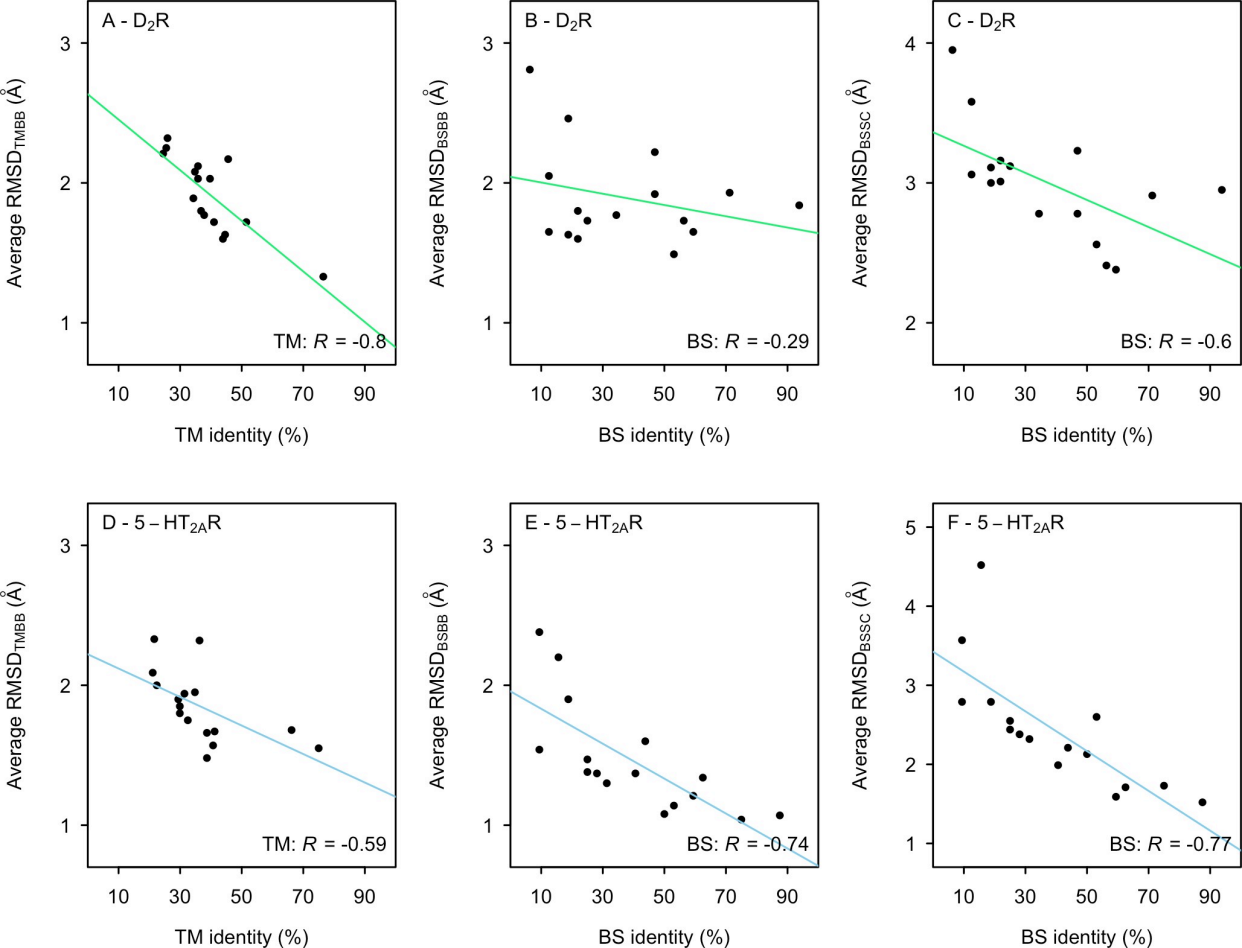
Relation between structural accuracy and sequence identity. The average RMSD_TMBB_ (A and D), RMSD_BSBB_ (B and E), and RMSD_BSSC_ (C and F) to the crystal structures for 50 models of the D_2_R (A-C) and 5-HT_2A_R (D-F) based on different templates. The solid line represents a linear regression and R is Pearson’s correlation coefficient.

The availability of crystal structures of the D_2_R [55] and 5-HT_2A_R [56] allowed us to evaluate the accuracy of the homology models. The average RMSDs to the experimental structure for the TM (RMSD_TMBB_), BS backbone (RMSD_BSBB_) and BS side chains (RMSD_BSSC_) were calculated. Although the resolutions of the crystal structures were not high enough to distinguish all side chain atoms in the electron density maps, RMSD_BSSC_ was calculated to assess if the overall shape of the binding pocket was captured (S3 Table and S1 Fig). There was a marked average improvement of structural accuracy if the templates with low (<30%) and high (>50%) sequence identities were compared whereas the results varied in the 30–50% range (Fig 1). The RMSD_TMBB_ had a moderate correlation with the TM sequence identity for both targets (R = –0.80 and –0.59 for the D_2_R and 5-HT_2A_R), respectively (Fig 2). A trend towards more accurate BS models for templates with higher sequence identity was obtained for the 5-HT_2A_R (R = –0.74 and –0.77 for RMSD_BSBB_ and RMSS_BSSC_, respectively). In contrast, relatively small improvements in binding site accuracy were obtained for the D_2_R with increasing sequence identity (R = –0.29 and –0.60 for RMSD_BSBB_ and RMSS_BSSC_, respectively). The weaker correlations were largely due to the fact that the D_3_R and D_4_R templates (71% and 94% BS sequence identity) did not have better RMSD values than 5-HT_2C_R and adrenergic receptor templates with 50–60% BS sequence identity. This result was supported by visual inspection of the models, which showed that the serotonin and adrenergic receptor templates led to better predictions of TM6 in the binding site region compared to the dopamine receptor structures.

### Molecular docking and ligand enrichment by homology models and crystal structures

Molecular docking screens of known ligands were carried out to further evaluate the homology models. For each of the 16 templates, 50 models were prepared for docking with DOCK3.6 [57,58]. A total of 822 and 650 known ligands of the D_2_R and 5-HT_2A_R combined with property-matched decoys [59] were docked to the homology models. Each compound was sampled in thousands of orientations in the binding sites, which were held rigid in the calculations. Successfully docked compounds were ranked by their predicted binding energy using a physics-based scoring function [57]. In total, the structures of >80 million complexes were predicted to evaluate virtual screening performance of the models. Enrichment of ligands over decoys was analyzed based on receiver operating characteristic (ROC) curves and quantified using the adjusted logarithm of the area under the curve (aLogAUC). The aLogAUC favors early enrichment, which is desirable in virtual screening, and positive values indicate that the docking scoring function performs better than random selection. For example, an aLogAUC value of 10 corresponds to identifying more than twice the number of ligands than expected from random selection [57]. To classify our enrichment results, we defined aLogAUC values of <10, 10–15, >15–20, and >20–25, and >25 as poor, fair, good, very good, and excellent, respectively.

**Fig 3:**
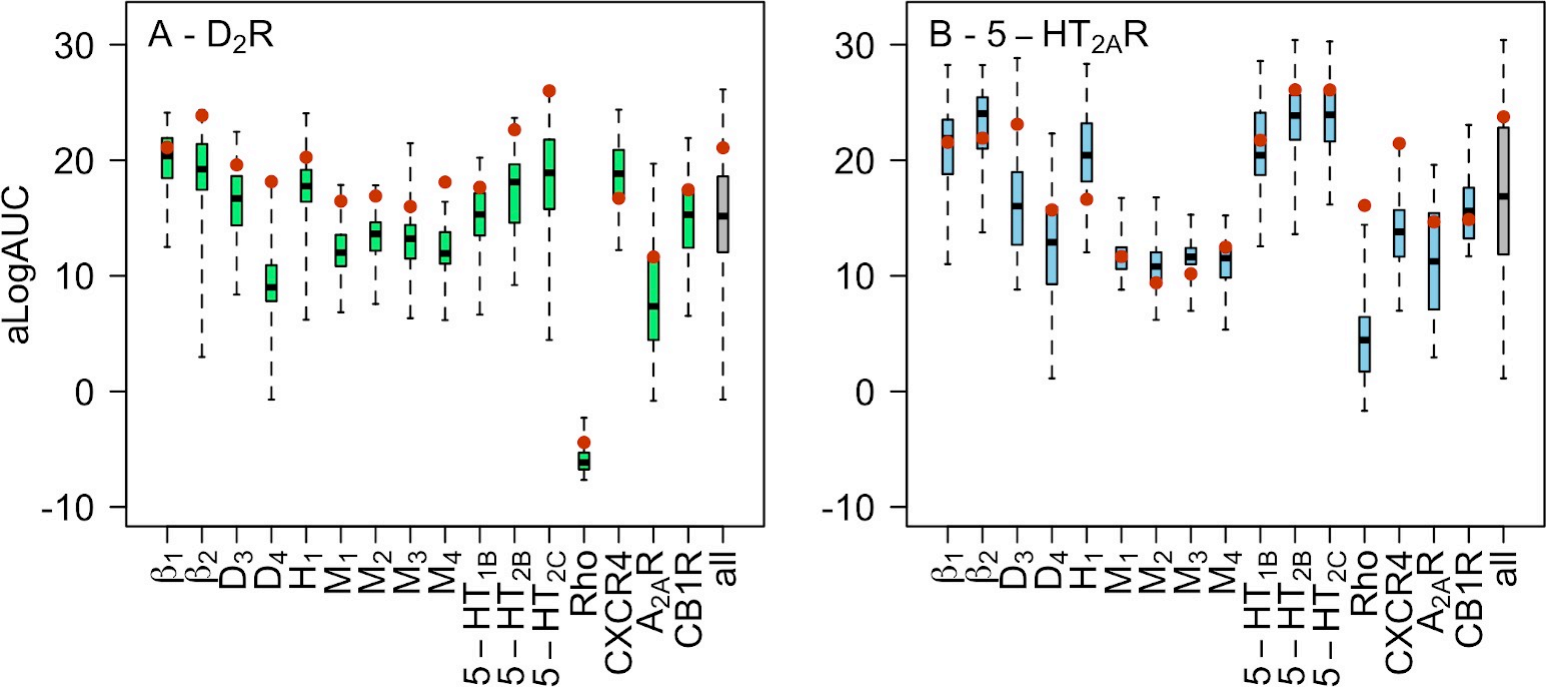
Ligand enrichment by homology models. Distribution of aLogAUC values for (A) D_2_R and (B) 5-HT_2A_R models based on 16 different templates. Distributions are shown using a boxplot representation. Each boxplot describes the results for 50 models obtained based on one template. The box represents the 50th percentile of the data and the black band shows the median value. The lowest and highest aLogAUC values are represented by the whiskers. The gray boxplot corresponds the results of all 600 models based on aminergic receptor templates. Ensemble enrichments are represented by red filled circles.

There was a large variation in the shapes of the ROC curves and aLogAUC values (S2 Fig and S4 Table), reflecting differences in the predicted structures of the binding sites. Even for the models based on the closest templates, the differences in aLogAUC between the best and worst models were 14 units and ligand enrichments ranged from poor to excellent. The template Rho resulted in a large number of models with enrichments close to or worse than what would be expected from random selection. For all the other templates, at least one model with ligand enrichment better than random selection was identified. The homology models were analyzed based on the median and maximal aLogAUC values, which are presented in Fig 3 and S4 Table. The median enrichment was taken as a measure of the quality of a template and the model with the highest aLogAUC value was assumed to be the best representative of the receptor structure. Good to very good median enrichments were obtained for seven out of the 12 aminergic templates. Of the five aminergic templates that resulted in fair ligand enrichments, four were based on muscarinic receptor structures, which shared relatively low sequence identities with the targets. Poor (D_2_R) to fair (5-HT_2A_R) median ligand enrichments were also obtained for the D_4_R template. The worst virtual screening performance was obtained among the models based on four non-aminergic templates. Two (CXCR4 and CB1R) and one (CB1R) template resulted in good median enrichment for the D_2_R and 5-HT_2A_R, respectively. The other distant templates either resulted in poor or fair ligand enrichments.

To further assess the quality of the models, we analyzed the best enriching structures for selected templates. For the aminergic templates, the best enriching models showed at least good enrichment and eight out of 12 models had very good to excellent enrichments. There were also structures with good to very good maximal enrichments among the screened CXCR4-, CB1R-, and A_2A_AR-based models, suggesting that these would be useful in virtual screening. The predicted binding modes of the 50 highest-ranked known ligands were inspected for the best-enriching models based on D_3_R, 5-HT_2C_R, CXCR4, A_2A_AR, and CB1R (Table 2). The binding modes were classified as good if the ligands formed a salt bridge to the conserved Asp^3.32^ (superscripts refer to Ballesteros-Weinstein residue numbering [60]) and extended towards TM5/6 with an aromatic moiety. It should be noted that these binding mode features are not only present in the D_2_R and 5-HT_2A_R crystal structures, but are conserved in all experimentally determined structures of aminergic GPCRs [61]. For the homology models based on the closest aminergic templates, 98–100% of the top-ranked ligands were docked in reasonable binding modes. In contrast, only the D_2_R model based on CXCR4 resulted in a high percentage of good ligand binding modes (96%) among the non-aminergic templates. For the other models based on non-aminergic templates, the predicted binding modes were judged to be poor with only 0–15% good poses. In fact, despite that there were D_2_R models based on the A_2A_AR with very good enrichment, the ligand binding site was not correctly identified. A majority of the top-ranked ligands formed a salt bridge to Glu95^2.65^ instead of the conserved Asp^3.32^. Similarly, in the case of the best enriching 5-HT_2A_R models based on CB1R, the salt bridge to Asp^3.32^ was captured, but the ligands extended towards a pocket formed by TM2 and TM7 instead of TM5.

**Table 2:** Assessment of overall binding modes of the 50 top-ranked ligands for homology models based on different templates and the crystal structures.

	Quality of ligand binding modes^a^			
	D_2_R		5-HT_2A_R	
Template	Good	Bad	Good	Bad
Aminergic^b^	50	0	49	1
CXCR4	48	2	7	43
A_2A_AR	0	50	2	48
CB1R	1	49	1	49
Crystal structure	48	2	49	1

^a^ Binding modes that captured a charge-charge interaction with Asp^3.32^ and extended towards TM5 with an aromatic moiety were classified as “Good”. Binding modes that failed to fulfil one of these criteria were classified as “Bad”.

^b^ Template with the highest TM and BS sequence identity to the D_2_R (D_3_R) and 5-HT_2A_R (5-HT_2C_R).

Docking screens were also carried out against crystal structures of the D_2_R and 5-HT_2A_R (S5 Table). The D_2_R screen yielded an excellent ligand enrichment (aLogAUC = 26.6) that outperformed the enrichment scores of all the 800 evaluated homology models. One of the 5-HT_2A_R crystal structures also yielded excellent virtual screening performance (aLogAUC = 26.1), but the enrichment was slightly lower than the maximum enrichment obtained for homology models based on the 5-HT_2C_R and 5-HT_2B_R templates. Similar to homology models based on closely related templates, close to 100% of the top-ranked ligands had reasonable binding modes in the crystal structures (Table 2).

As an additional control, we also docked the D_2_R and 5-HT_2A_R ligands to the template crystal structures used to generate the homology models. In previous studies, template structures and homology models were found to enrich ligands equally well [32,62]. The calculated ligand enrichments by the templates are presented in S6 Table. A homology model that performed better than the corresponding template was identified for 14 and 16 out of the 16 sets of D_2_R and 5-HT_2A_R models, respectively. A few template structures enriched ligands very well, *e*.*g*. adrenergic and serotonin receptors. The result can be explained by that these receptors also recognize biogenic amines and are hence likely to bind many D_2_R and 5-HT_2A_R ligands. Large improvements of ligand enrichment were obtained for some of the distant templates, *e*.*g*. the muscarinic receptors and CXCR4.

### Accuracy of ECL2 and its impact on ligand enrichment

One of the most challenging aspects of GPCR modeling is to predict the structure of ECL2, which is part of the orthosteric site and has varying length and diverse sequences[15–17,63]. Whereas the ECL2 of the 5-HT_2A_R was well predicted by several templates (*e*.*g*. 5-HT_2C_R), the D_2_R loop had a unique shape that was not captured by any available crystal structure (S3 Fig). To investigate if a more accurate loop could improve ligand enrichment, we generated a new set of D_2_R models based on the D_3_ subtype with the structure of ECL2 extracted from the D_2_R crystal structure. This model hence had a perfect structure of ECL2, and the TM region was based on the most closely related template. The resulting models had a median aLogAUC of 23.6, which was 7.0 aLogAUC units better than the models based on the D_3_R crystal structure and close to that of the D_2_R crystal structure (aLogAUC = 26.6).

Early studies of GPCR homology modeling discovered that virtual screening performance could be insensitive to or even improved by excluding ECL2 from the calculations [63]. To assess this option, we rescreened all homology models in the absence of ECL2. The median aLogAUC values were either comparable to or higher than those obtained with the loop present (S7 Table). The largest differences were found for D_2_R models based on the D_3_R, D_4_R, and Rho templates, which all performed substantially better if ECL2 was excluded. The Rho template positioned the D_2_ loop in a region where it blocked accurate positioning of ligands, which explained the improved results in this case. To further assess the quality of the predicted complexes, ligand binding modes were analyzed for the D_2_R and 5-HT_2A_R models based on the closest aminergic template. For the D_2_R and 5-HT_2A_R models with the best ligand enrichments, the binding modes of docked ligands were judged to be equally well predicted in the presence (Table 2, 98–100% good binding modes) and absence (100% good binding modes) of ELC2.

### Relation between ligand enrichment, sequence identity, and structural accuracy

**Fig 4:**
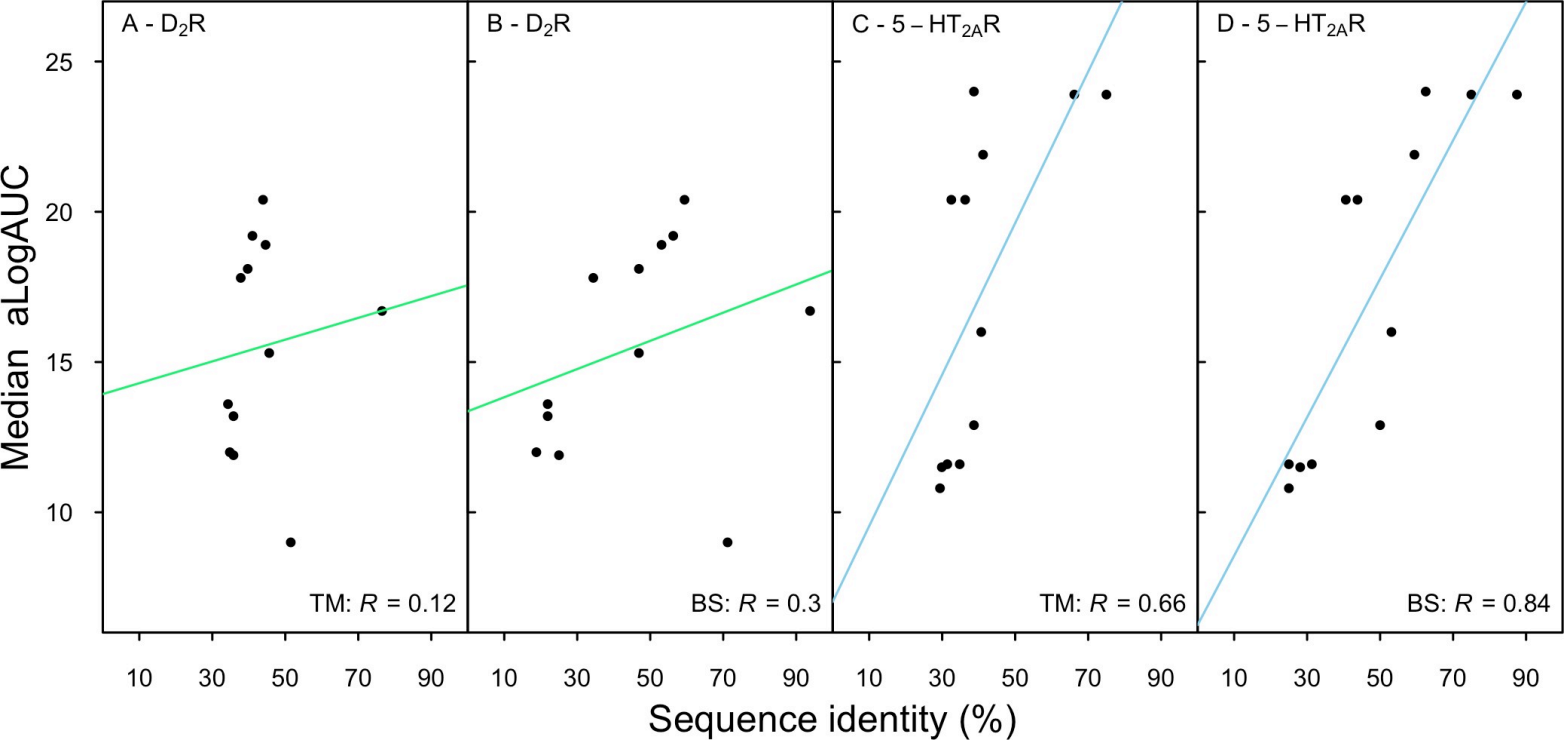
Relation between ligand enrichment and sequence identity. The median aLogAUC values of the D_2_R (A-B) and 5-HT_2A_R (C-D) homology models based on aminergic templates with different TM (A and C) or BS (B and D) sequence identities. The solid line represents a linear regression and R is Pearson’s correlation coefficient.

**Fig 5:**
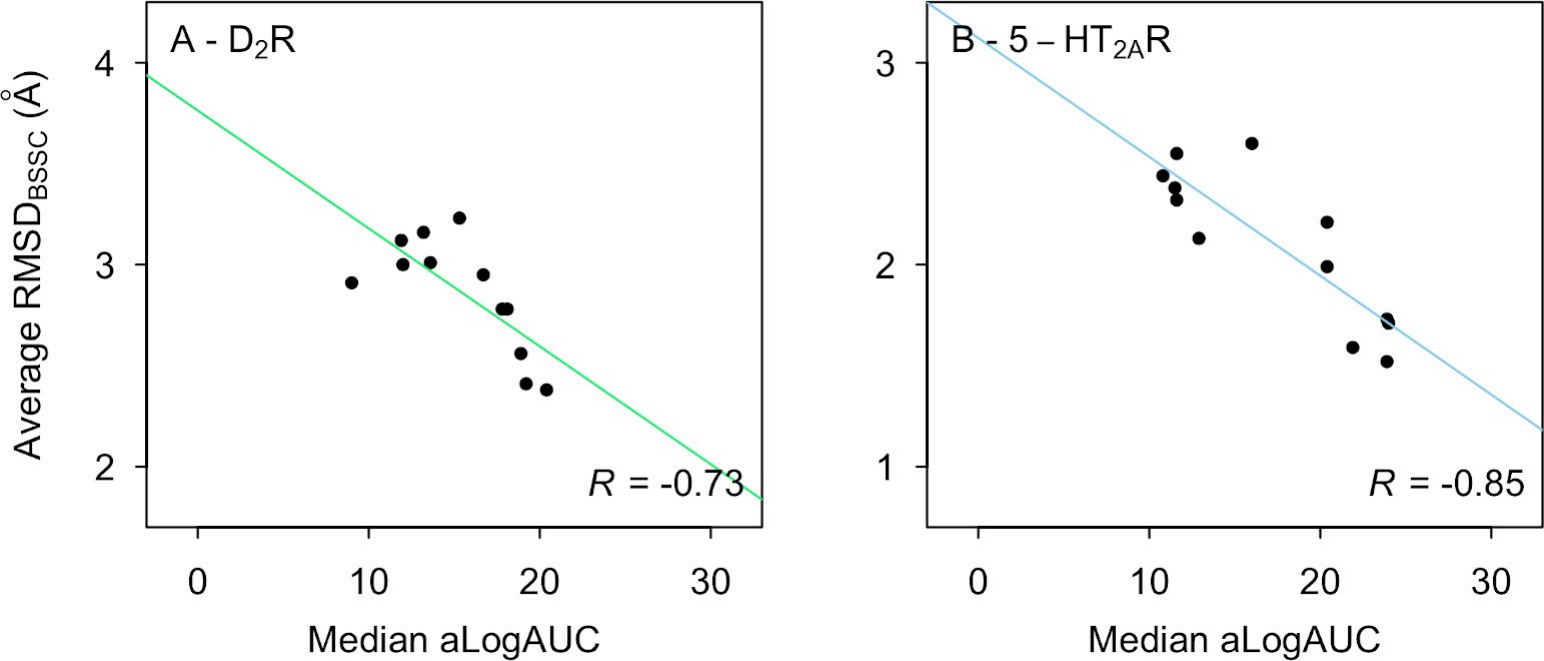
Relation between ligand enrichment and structural accuracy. The median aLogAUC and average RMSD_BSSC_ to the crystal structures for 50 homology models of the D_2_R (A) and 5-HT_2A_R (B) based on aminergic templates. The solid line represents a linear regression and R is Pearson’s correlation coefficient.

We assessed if the median ligand enrichments displayed by the homology models correlated with sequence identity or structural accuracy. Only the aminergic templates were included in this analysis because the non-aminergic templates generally did not produce reasonable ligand binding modes. For the D_2_R, no correlation was found between sequence identity and aLogAUC whereas moderate correlation was found for TM (R = 0.66) and BS (R = 0.84) regions of the 5-HT_2A_R (Fig 4). The lack of correlation for the D_2_R was partly due to that the models based on the D_3_ and D_4_ subtypes yielded surprisingly low ligand enrichments. However, it should be noted that this result was consistent with the fact that both the binding site structures (Fig 2B and 2C) and ECL2 (S3 Fig) were relatively poorly predicted by these templates. For example, the 5-HT_2C_R and adrenergic receptor templates with 50–60% BS sequence identity resulted in more accurate binding site models and also yielded better ligand enrichment. Interestingly, removing ECL2 (S4 Fig) significantly improved the correlation between ligand enrichment and sequence identity for the D_2_R models (R = 0.67 and 0.84 for TM and BS, respectively) whereas the results for the 5-HT_2A_R were unaffected (R = 0.66 and 0.89 for TM and BS, respectively). Finally, we evaluated the relationship between ligand enrichment and structural accuracy (Fig 5) and found a moderate correlation between RMSD_BSSC_ and aLogAUC for both receptors (R = –0.73 and –0.85 for D_2_R and 5-HT_2A_R, respectively).

### Ligand enrichment by ensembles of homology models

An alternative to using a single rigid structure in docking screens is to consider an ensemble of models, which could account for binding site plasticity. The ensemble enrichment was calculated by identifying the best docking score of each docked compound among multiple homology models, leading to a single aLogAUC value for the entire set. The ensemble enrichments were calculated for the aminergic templates by considering the 50 homology models generated in each case and the combined set of 600 homology models (Fig 3 and S4 Table).

For the D_2_R, the ensemble enrichments were consistently better than the medians of the individual models. Templates resulting in models with good median enrichments had very good or excellent ensemble enrichments, and good ensemble enrichments were obtained for models with poor or fair median enrichments. For 10 out of 12 templates, the ensemble enrichments were even comparable to the maximal individual enrichments, but there was no significant improvement over the best individual models. For example, the models of the D_2_R based on the D_4_R crystal structure resulted in poor performance (median aLogAUC = 9.0) whereas the ensemble had good ligand enrichment (aLogAUC = 18.1), which was similar to the maximal enrichment of the individual models (aLogAUC = 17.8). Finally, the ensemble enrichment for all D_2_R models based on aminergic templates was very good (aLogAUC = 21.1), which was close to five aLogAUC units greater than the median of this set. For the 5-HT_2A_R, the ensemble enrichments were very good to excellent for six templates. However, in contrast to the results for the D_2_R, the ensemble enrichments did not reach values comparable to the best individual models. Instead, ensemble aLogAUC values were close to the medians for 10 templates and significantly improved over the median only in one case. The ensemble enrichment for all the 5-HT_2A_R models was very good (aLogAUC = 23.8), which was better than the median ligand enrichment of this set.

### Influence of template on enrichment of ligand chemotypes

**Fig 6:**
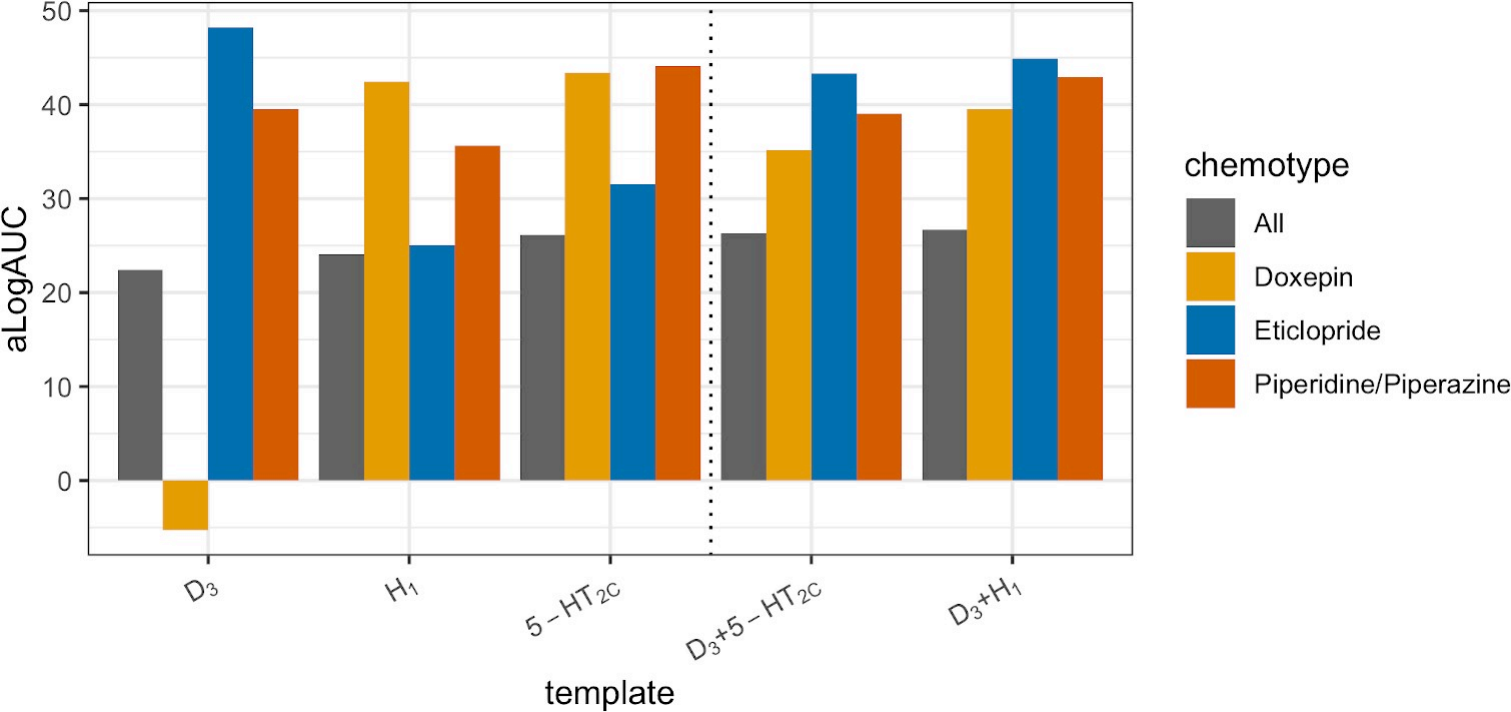
Influence of template on enrichment of ligand chemotypes. Enrichment (aLogAUC) of eticlopride- (blue bars), doxepin- (yellow bars), piperidine/piperazine-like (red bars), and all (grey bars) D_2_R ligands by D_2_R homology models. Homology models based on three different templates (D_3_R, H_1_R, and 5-HT_2C_R) and ensemble enrichments of the D_3_R model combined with either the H_1_R and 5-HT_2C_R models were evaluated.

A potential concern regarding the use of a static homology model in virtual screening is that the binding site conformation of the template could bias the results towards specific chemotypes, in particular compounds similar to the co-crystallized ligand. Such effects were analyzed in detail for homology models of the D_2_R. D_2_R ligands similar to eticlopride and doxepin were identified as these ligands were bound in the D_3_R and H_1_R templates, respectively [39,41]. A set of piperidine/piperazine-like ligands were also collected as this is a privileged scaffold for aminergic receptors and a representative compound (ritanserin) was co-crystallized in the 5-HT_2C_R template [44]. The three sets of compounds (S8 Table) and decoys were docked to D_2_R models based on the D_3_R, H_1_R, and 5-HT_2C_R templates. The calculated aLogAUC values indicated a bias toward chemotypes similar to the co-crystallized ligands (Fig 6). For each template, the best ligand enrichment was obtained for the set of compounds similar to the co-crystallized ligand and the largest differences were found for the eticlopride- and doxepin-like ligands. Doxepin-like compounds were not identified by the D_3_R-based model (aLogAUC = –5.3), but the enrichment of the same set of compounds by the H_1_R model was excellent (aLogAUC = 42.4). Similarly, the enrichment of eticlopride-like ligands was stronger by the D_3_R-based model (aLogAUC = 48.3) than by the H_1_R-based (aLogAUC = 25.0). To investigate if chemotype bias could be alleviated by considering an ensemble of structures, we combined the model based on the D_3_R with either the H_1_R or 5-HT_2C_R-based models. The ensembles based on two templates led to strong enrichment of all three chemotypes and also slightly improved the enrichment of the full set of ligands compared to the D_3_R-based model.

### MD simulation refinement of homology models

MD simulations of homology models based on close and distant templates were performed to explore if structural accuracy and virtual screening performance could be further improved. MD simulations of D_2_R and 5-HT_2A_R models based on D_3_R and 5-HT_2C_R, respectively, were carried out for the apo form and in complex with the co-crystallized ligands of the templates, which were active at the both the targets and templates [64,65]. For the Rho-based models, only the apo form was considered. Each system was equilibrated in a hydrated lipid membrane and three simulation replicates of 100 ns were generated.

MD simulation snapshots were first compared to the homology model used as starting structure. For the models based on closely related templates, the average RMSD_TMBB_ values were 1.3–1.9 Å and 1.7–2.2 Å for the simulations of the D_2_R and 5-HT_2A_R, respectively (S9 Table and S5 Fig). The Rho-based models drifted further away from the homology models with average RMSD_TMBB_ values of 2.3–2.4 Å and 3.2–3.4 Å for D_2_R and 5-HT_2A_R, respectively (S9 Table and S5 Fig). Visual inspection of simulation snapshots showed that the overall topology of the receptors was maintained with no large conformational changes in the TM region. In the 5-HT_2A_R simulations, unfolding of a few residues at the intracellular tips of TM5 and TM6 simulations was observed, which may be attributed to that intracellular loop three was not included in the simulations. In simulations of the receptor-ligand complexes, the binding modes of eticlopride and ritanserin were maintained throughout the simulation.

**Fig 7:**
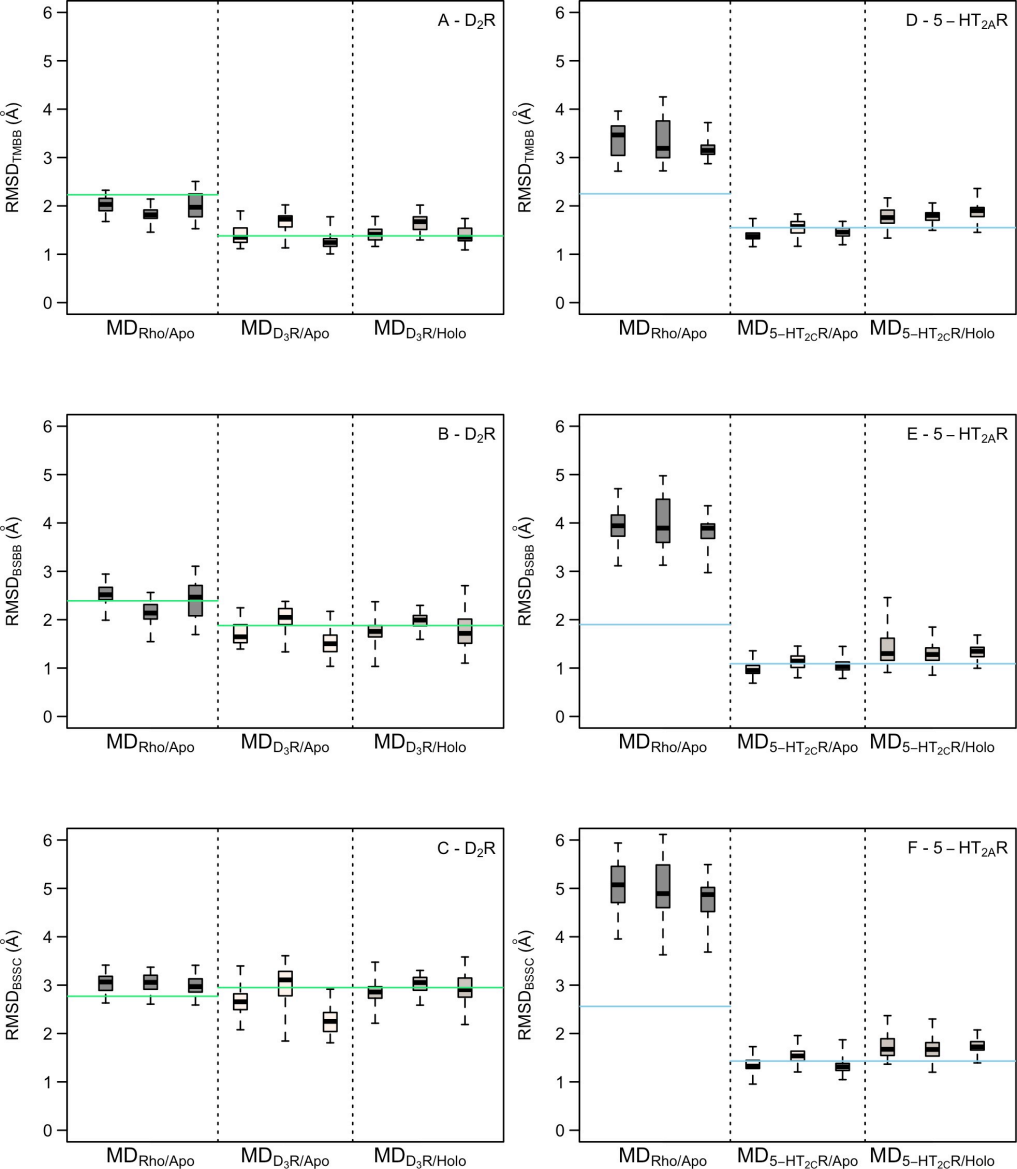
Structural accuracy of MD simulation snapshots. RMSD distribution of the TM backbone, BS backbone, and BS side chains of MD snapshots to the crystal structure for the D_2_R (A-C) and 5-HT_2A_R (D-F). Distributions of RMSD values for the three sets of snapshots based on the Rho-based models (MD_Rho/Apo_) and homology models based on the most closely related template in apo (MD_Template/Apo_) and holo forms (MD_Template/Holo_) are shown using a boxplot representation. The box represents the 50th percentile of the data and the black band shows the median value. The lowest and highest RMSD values are represented by the whiskers. The horizontal lines show the RMSD values of the homology model used as starting structure.

Each MD simulation trajectory was clustered and 50 snapshots (cluster centers) were compared to the corresponding crystal structure of the target receptor (Fig 7). For the D_2_R models based on the D_3_ subtype, the RMSD_TMBB_ to the crystal structure were similar for the apo and holo forms with median values ranging from 1.2 to 1.7 Å for the six trajectories. For comparison, the D_2_R homology model used as starting structure had a corresponding RMSD_TMBB_ of 1.4 Å. Overall, 44%, 58%, and 62% of the MD-refined structures had improved TM backbone, BS backbone, and BS side chain RMSD values compared to the homology model, respectively. Inspection of the snapshots confirmed that the TM region remained close to the initial homology model, but there was a larger variation in ECL2, which reduced the volume of the binding site. The Rho-based homology model of the D_2_R had an RMSD_TMBB_ to the crystal structure of 2.2 Å. The corresponding median RMSD values for the MD snapshots were 1.8–2.0 Å. In this case, 87%, 50%, and 11% of the MD-refined structures had improved TM backbone, BS backbone, and BS side chain RMSD values compared to the homology model, respectively. The improved accuracy was due to rearrangement of TM6 in the vicinity of the binding site. However, inspection of the models also showed that TM3 was shifted inward and blocked access to part of the binding site occupied by aminergic ligands. For the 5-HT_2A_R, the median RMSD_TMBB_ to the crystal structure ranged from 1.4 to 1.9 Å for the simulations based on the 5-HT_2C_R homology model, which had an RMSD_TMBB_ of 1.6 Å. The binding site, which was very well predicted by the homology model, slightly diverged from the crystal structure in the holo simulation and was maintained for the apo form. Overall, 38%, 37%, and 32% of the MD snapshots showed improved agreement with the crystal structure for the TM backbone, BS backbone, and BS side chains, respectively. The Rho-based 5-HT_2A_R homology model drifted substantially further away from the crystal structure with median RMSD_TMBB_ values of 3.1–3.5 Å, which can be compared to 2.3 Å for the homology model. None of the MD-refined structures were better than the initial homology model in this case.

**Fig 8:**
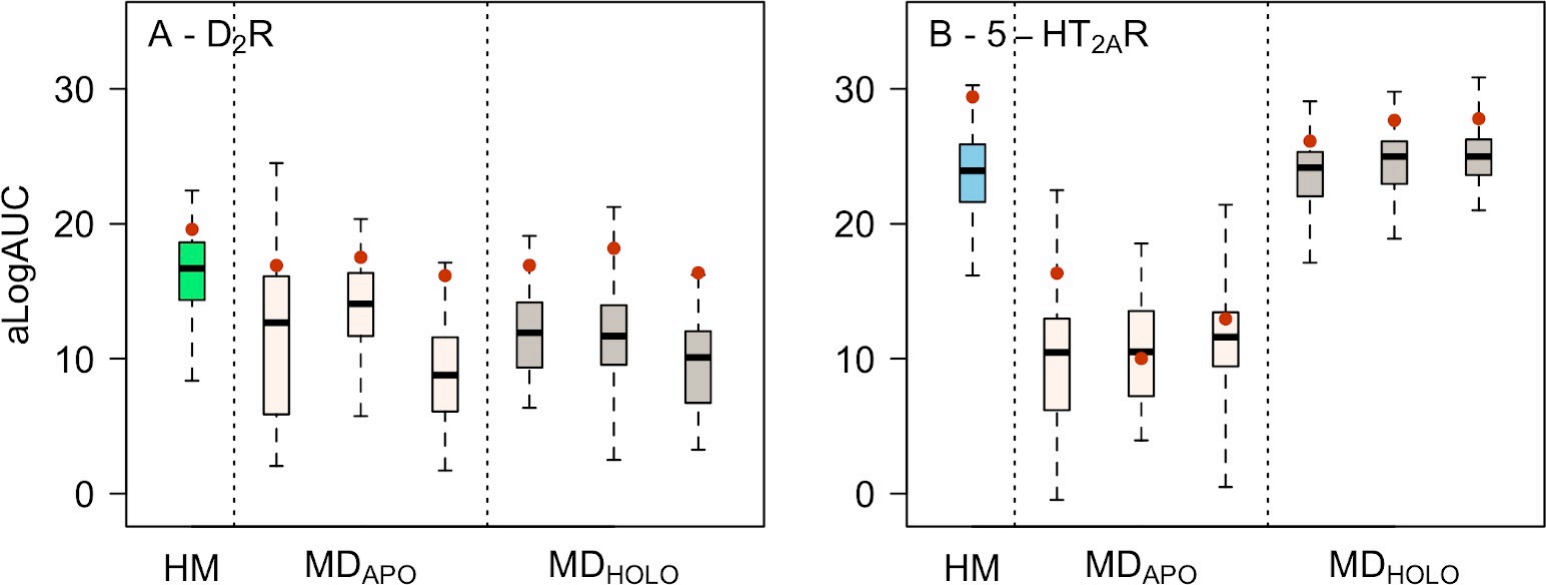
Ligand enrichment by MD simulation snapshots. Distributions of aLogAUC values for 50 homology models (HM) and sets of 50 snapshots from three MD simulations per template for the (A) D_2_R based on the D_3_R template and (B) 5-HT_2A_R based on the 5-HT_2C_R template. Results for HM, apo (MD_APO_), and holo forms (MD_HOLO_) are shown using a boxplot representation. The box represents the 50th percentile of the data and the black band shows the median value. The lowest and highest alogAUC values are represented by the whiskers. The ensemble enrichments of each set of 50 models are represented by red filled circles.

Molecular docking of ligands and decoys were carried out against the MD snapshots to compare their virtual screening performance to the homology models (S10 Table). The MD-refined homology models based on the Rho template were not assessed because the orthosteric binding site had either collapsed (D_2_R) or clearly drifted far away from the crystal structures (5-HT_2A_R). The median ligand enrichments ranged from poor to fair for the six sets of snapshots of the D_2_R. The median enrichments were consistently worse than for the homology models based on the same template (Fig 8), which could be explained by the large variation in ECL2 among the snapshots. The best performing MD-refined structures had enrichments that were comparable to that of the best homology model. For the 5-HT_2A_R, the median ligand enrichments for the MD snapshots ranged from fair to very good (Fig 8). The best median and maximal ligand enrichments were obtained if the ligand was present in the simulation and, in this case, the results were comparable to that of the homology models based on the same template. In the snapshots from the apo simulation, side chains partially blocked the binding site, resulting in worse ligand enrichment than the homology models. In agreement with the results obtained for sets of homology models, ensemble enrichments did not outperform the best individual MD snapshots. Instead, the results were either comparable to the best model (D_2_R) or the median (5-HT_2A_R).

## Discussion

Homology modeling has the potential to bridge the gap between the large number of GPCRs in the human genome and the few experimentally determined structures available to study these at atomic resolution. We evaluated the performance of structure-based virtual screening against homology models based on different templates and strategies for treating binding site plasticity. Four main findings emerged from the modeling of two drug targets from the GPCR family and extensive molecular docking screens against predicted receptor structures. First, our results generally agreed with the notion that selection of a template with higher sequence identity will lead to better models. However, the correlation between structural accuracy and sequence identity was not particularly strong because the best binding site model was not necessarily based on the closest homolog. Second, molecular docking can be used to identify models that perform well in virtual screening and ligand enrichment increased with binding site accuracy. Homology models based on distant templates (<30% sequence identity) are in most cases not suitable for virtual screening because of modeling errors in the binding site, which makes it difficult to obtain accurate predictions of receptor-ligand complexes. Third, consideration of binding site plasticity by screening ensembles of structures did generally not improve virtual screening performance, but in favorable cases, the results were comparable to the best individual model. Finally, MD refinement led to moderate improvements of structural accuracy in some cases, but virtual screening performance of simulation snapshots were either comparable to or worse than that of homology models.

A widespread assumption is that the template with the highest sequence identity to the target will result in the best homology model. To assess this rule of thumb, we analyzed relationships between sequence identity, binding site accuracy, and ligand enrichment. There was a large variation in ligand enrichment by homology models even if binding site structures were generated based on the same template. Large sets of models with different side chain conformations must hence be considered to evaluate the quality of a template. This finding makes it difficult to compare our results to previous studies that were mainly based on a single homology model per template [24,32,34]. In agreement with expectations, the most accurate predictions of the 5-HT_2A_R binding site were obtained based on closely related 5-HT_2_ subtypes (66–75% TM sequence identity) and these models showed excellent virtual screening performance. However, there were also templates with lower TM sequence identity (~40%) that resulted in comparable structural accuracy and enrichment. In contrast, the best models of the D_2_R binding site were not based on the other D_2_-like receptors (D_3_R and D_4_R). Instead, there were templates with TM sequence identities in the 40–45% range that resulted in better representations of the binding site and these models also performed well in virtual screening. This result is consistent with a previous study comparing the virtual screening performance of D_2_R models based on the β_2_AR and D_3_R templates using a different docking software and ligand sets [21]. Notably, the best homology models were accurate structurally and yielded ligand enrichments that were comparable to or even better than those obtained using the D_2_R and 5-HT_2A_R crystal structures. Our observations lead to new guidelines for GPCR modeling. If templates with >30% sequence identity are available, several of these should be evaluated in retrospective virtual screens. All templates with >50% TM sequence identity need to be considered. In addition, the templates with the highest binding site sequence identity should be prioritized from the 30–50% range. As template performance could be influenced by chemotype-bias, structures in complex with different ligands should also be explored if such are available. Finally, multiple models per template should be evaluated in retrospective docking screens, and the binding structures that enrich known ligands well will be suitable for prospective screening.

Homology modeling based on templates with <30% sequence identity can occasionally yield models that show good enrichment and reasonable ligand binding modes, *e*.*g*. in the case of the CXCR4-based model of the D_2_R. However, in most cases accurate prediction of ligand binding sites appears to be out of reach for homology modeling based on distant templates. The backbone structure and ECL2 of the template are likely to deviate from the target, which will often lead to poor predictions of receptor-ligand complexes. An illustrative prospective example of the difficulties to make accurate predictions based on distant templates is the homology models of CXCR4 constructed by Mysinger *et al*. prior to the release of the crystal structure. Excellent retrospective ligand enrichment was achieved, but the subsequently released crystal structure revealed that the ligands were docked to the wrong binding site [12]. Better docking scoring functions as well as accurate representation of backbone and side chain flexibility will be necessary to improve models of GPCR-ligand complexes.

When only distant templates are available or low ligand enrichments are obtained using templates with high sequence identity, the structure of ECL2 may be poorly predicted. For example, we found that the ECL2 of the D_2_R was different from the other subtypes and improving the accuracy of the loop improved ligand enrichment. If the accuracy of the ECL2 is uncertain, one approach is to simply exclude it from the model in the docking calculations [63]. Interestingly, ligand enrichment was then improved or maintained for a majority of the templates. However, considering the structural diversity of ECL2, this approximation is unlikely to be generally valid. Loop modeling hence remains to be one of the major challenges of GPCR structure prediction. As *ab initio* prediction of loops is very difficult, the best approach for modeling of ECL2 may be to utilize information from multiple templates, *e*.*g*. by extracting the TM and loop regions from different receptors, which was successful in blind predictions of GPCR structures [13,18].

Another important aspect of template selection and evaluation, which was not investigated in this work, is the activation state of the target GPCR. Receptor activation involves a large movement of TM6 and a contraction of the orthosteric binding site relative to the inactive conformation [66]. If structures of a template have been determined in several states, homology modeling should be performed based on the conformation that is most relevant for the goal of the virtual screen. In such cases, model evaluation by molecular docking screening can also be focused on specific ligand sets, *e*.*g*. agonists or antagonists [67,68].

Treatment of binding site plasticity is one of the major methodological challenges in molecular docking. One approach to incorporate a flexible representation of the binding site is to screen ensembles, which can be composed of experimentally determined structures (*e*.*g*. from crystallography or NMR) or be generated computationally (*e*.*g*. by MD simulations, normal mode analysis, or homology modeling) [69]. In this work, we explored the use of homology model ensembles derived from one or several templates as well as ensembles of MD snapshots. Our finding that ensembles rarely outperform the best single model agrees with results obtained in other benchmarks [32,34]. However, it should be noted that there are several scenarios where it can be advantageous to use ensembles of models in prospective screens. First, docking to ensembles is suitable for targets with few or no known ligands. In such cases, the best models cannot be identified based on ligand enrichment. Our results suggest that the ensemble enrichment will at least be comparable to the median of the individual models and, in favorable cases, could have similar performance as the best model. Second, we showed that consideration of ensembles based on different templates can reduce bias towards certain ligand chemotypes, which can increase the diversity of hits from docking screens. Finally, as demonstrated by previous prospective studies [26,28], it will be essential to consider binding site flexibility if the aim of the screen is to identify selective ligands by docking to targets and antitargets. In such applications, a flexible representation of the receptor is crucial to ensure that predicted ligands do not bind to any accessible conformation of the antitarget.

Inherent limitations of homology modeling can lead to errors in a predicted structure that could potentially be corrected by refinement with higher-level methods. Even if templates with high sequence identity were available for the D_2_R and 5-HT_2A_R, there were local deviations between homology models and crystal structures that influenced virtual screening performance. Differences between the relative orientation of the TM helices will increase with decreasing sequence identity, which will cause errors in the shape of the binding site. Given an accurate force field energy function and sufficient simulation time, MD should make it possible to generate relevant conformations of the binding site. Our results suggest that although MD-refinement can generate receptor conformations that are more similar to the native structure than the homology model, it is equally probable that the simulations drift further away from the crystal conformation. Although a larger test set and longer simulations would be required to quantify the potential of MD simulations to improve GPCR homology models, our results agree with previous studies of soluble proteins [70]. It should also be noted that MD protocols that use some restraints on the protein structure have been able to consistently improve homology modeling accuracy and will likely also be useful for GPCRs [71]. To compare virtual screening performance of homology models and MD snapshots, docking calculations for snapshots from apo and holo simulations were performed. Ligand enrichments by MD snapshots were either worse or comparable to the results obtained for homology models. One could argue that the ensembles from MD simulations may represent relevant conformational states of the receptors that were not captured by crystallography or homology modeling. In line with this idea, Vass *et al*. demonstrated that different ligand chemotypes were identified by homology models and MD simulation snapshots in a prospective docking screens against the H_4_R [31]. MD-refined structures can hence be useful in virtual screening to improve the diversity of hits, but considering the high computational cost of simulations and lack of improvement of retrospective ligand enrichment, homology modeling based on several different templates should be prioritized in GPCR structure prediction.

## Materials and methods

### Sequence alignment and homology modeling

A multiple sequence alignment (MSA) based on aminergic GPCRs from the UniProt database [72] and the template crystal structures (Table 1) was generated using the MAFFT localpair algorithm with default parameters [73]. For this MSA, an HMM profile was obtained using hmmbuild from the HMMER suite [74]. The resulting alignment was manually adjusted if gaps were present in the TM region and in the extracellular loop regions. TM regions of the D_2_R and 5-HT_2A_R were defined based on well aligned regions in the MSA and structural information available for aminergic receptors (S1 Table). The conserved cysteine in ECL2 and the two following amino acids of the template and target were aligned. Prior to homology modeling, the N- and C- termini, intracellular loop 3, and the stretch of ECL2 between TM4 and the conserved cysteine in ECL2 were excluded from the alignment. Homology models were built using MODELLER (version 9.14) [53]. A total of 250 models were generated per template-target pair, and the 50 models with the best DOPE scores [54] were extracted for further analysis. RMSD calculations, which accounted for side chain symmetry, for the homology models and crystal structures were performed using PyMol [75]. All statistical analyses were performed with R 3.6.1 (https://www.r-project.org).

### Preparation of known ligands and decoys

Sets of known D_2_R and 5-HT_2A_R ligands (activity < 1 μM and molecular weight < 350 Da) were extracted from ChEMBL20 [76]. The molecular weight range was selected in order to focus the benchmarking set on compounds with similar properties to those present in chemical libraries used in virtual screening, e.g. the ZINC database [77]. No filtering based on the functional activity of the ligands was performed. Property-matched decoys to the D_2_R and 5-HT_2A_R ligands (822 and 650 unique ligands with 55146 and 43777 decoys, respectively) were obtained using the DUD-E approach (http://dude.docking.org/) [59]. To evaluate enrichments for specific ligand chemotypes, D_2_R ligands similar to the co-crystallized ligands of three templates (H_1_R/Doxepin: 45 ligands, D_3_R/Eticlopride: 38 ligands, and 5-HT_2C_R/Ritanserin: 48 ligands) were identified using clustering and substructure searches (S8 Table). Compounds were prepared for docking with DOCK3.6 using the ZINC database protocol [78].

### Molecular docking screens

Molecular docking calculations were performed using the DOCK3.6 program [57,58]. The receptor was described using a version of the AMBER force field. Parameters for the ionized side chains were used for all Asp, Glu, Arg, and Lys residues. Tautomers of His residues were selected by visual inspection of hydrogen bonding networks and were the same for all models of the D_2_R (Nε protonated: His33, His106, His393, and His398; Nδ protonated: His442) and 5-HT_2A_R (Nε protonated: His70, His165 and His182; Nδ protonated: His183). The binding site of the homology models was defined based on the co-crystallized ligand of the template. The binding sites of the D_2_R and 5-HT_2A_R crystal structures (PDB codes 6CM4 [55] and 6A94 [56], respectively) were defined based on the co-crystallized ligands. Compounds were docked to a rigid receptor structure using the DOCK3.6 flexible-ligand algorithm with 45 matching spheres, which were labelled for chemical matching. The number of generated orientations of the docked compound was determined by the bin size, bin size overlap, and distance tolerance, which were set to 0.4, 0.1, and 1.5 Å, respectively. The binding energy of each docked compound was calculated as a sum of the van der Waals interaction energy, electrostatics interaction energy, and a ligand desolvation energy term [57]. Enrichment of ligands over decoys was analyzed using a receiver operating characteristic (ROC) curve. To quantify the ligand enrichment, a semi-log transformation of the ROC curve was performed, followed by integration of the area under curve to obtain the LogAUC value. The adjusted logAUC (aLogAUC) is calculated by subtracting the logAUC value obtained from random selection [57].

### Molecular dynamics simulations

MD simulations were performed using the homology model with the best DOPE score [54]. In these calculations, intracellular loop three, N and C termini were excluded. N- and C-termini were capped with acetyl and methylamide groups, respectively. Protonation states of ionizable residues were determined with PropKa [79] except for His393 of the D_2_R and His182 of the 5-HT_2A_R models, which were protonated at Nε. A sodium ion was placed close to Asp^2.50^ in the TM region, which has been observed in inactive state structures of GPCRs [50]. The homology models were aligned to a GPCR of the same type in the Orientations of Proteins in Membranes (OPM) database [80] using STAMP 4.4 [81]. The receptors were then embedded into a POPC bilayer and solvated. The systems were built using HTMD tools [82]. Each system was neutralized with sodium chloride (0.15M) before undergoing 5000 conjugate gradient minimization steps. We used the CHARMM36m force fields for protein, lipids, and ions. We used the TIP3P model for water [83] and the RATTLE algorithm [84] to constrain bonds involving hydrogens. Ligand parameters were obtained using the CHARMM General Force Field (CGenFF) with the ParamChem webserver (legacy version 1.0.0) [85,86]. MD simulations were performed with ACEMD [87]. The systems were equilibrated for 40 ns at 310 K using a Langevin thermostat with a damping constant of 1 ps^-1^, a Berendsen barostat at 1 atm with a pressure relaxation 800 fs and a compressibility factor of 4.57x10^-5^ with a 2 fs time-step (NPT ensemble). During the first 20 ns of the equilibration, we applied harmonic position restraints (1.0 kcal mol^-1^ Å^-2^) to the protein backbone and the sodium ion coordinated by Asp^2.50^. The restraints were then gradually removed using a slope of -0.095 kcal mol^-1^ Å^-2^ ns^-1^ for 10 ns and fully removed for the last 10 ns. Production runs (three replicas) were performed in the NVT ensemble and 100 ns were generated with a time-step of 4 fs, using a hydrogen mass repartition scheme [88], at 310 K using a Langevin thermostat with a damping constant of 0.1 ps^-1^. Cutoffs for Lennard-Jones and short-range electrostatic interactions were set to 9 Å and a smooth switching function was applied when the distance exceeded 7.5 Å. Long-range electrostatic forces were calculated using the particle-mesh Ewald algorithm [89] with a grid spacing of 1 Å. The receptors were simulated both in apo forms and in complex with eticlopride and ritanserin for the D_2_R and 5-HT_2A_R, respectively. Coordinates were saved every 100 ps. MD trajectories were analyzed using the MDAnalysis [90,91] and MDTraj [92] packages. Clustering of the trajectories was performed based on the TM backbone using the Ward method in TTClust [93] and 50 diverse snapshots were selected for analyses.

## Supporting information

S1 Table Definition of the TM helix region using Ballesteros-Weinstein numbering. (PDF)

S2 Table Average pairwise RMSDs for the binding site side chains of the D_2_R and 5-HT_2A_R homology models. Statistics are based on 50 homology models per template.
(PDF)

S3 Table Average RMSDs of D_2_R and 5-HT_2A_R homology models to the crystal structures. Statistics are based on 50 homology models per template.
(PDF)

S4 Table Ligand enrichment (aLogAUC) by D_2_R and 5-HT_2A_R homology models based on different templates. Statistics are based on 50 homology models per template.
(PDF)

S5 Table Ligand enrichment (aLogAUC) by the D_2_R and 5-HT_2A_R crystal structures. (PDF)

S6 Table Ligand enrichment (aLogAUC) by crystal structure templates. (PDF)

S7 Table Ligand enrichment (aLogAUC) by D_2_R and 5-HT_2A_R homology models without ECL2. Statistics are based on 50 homology models per template.
(PDF)

S8 Table Smiles of D_2_R ligands similar to the co-crystallized ligands of three templates (doxepin-, eticlopride-, piperidine/piperazine-like ligands) from the ChEMBL database. (PDF)

S9 Table Average RMSDs to the starting structure (homology model) of MD snapshots of the D_2_R and 5-HT_2A_R. (PDF)

S10 Table Ligand enrichments (aLogAUC) for MD simulation snapshots of the D_2_R and 5-HT_2A_R homology models. Statistics are based on 50 snapshots per trajectory.
(PDF)

S1 Fig Binding site accuracy of homology models. Distributions of the RMSD_BSSC_ to the crystal structures for 50 models of the D_2_R (A) and 5-HT_2A_R (B) based on different templates using a boxplot representation. The box represents the 50th percentile of the data and the black band shows the median value. The lowest and highest RMSD_BSSC_ values are represented by the whiskers.
(TIF)

S2 Fig Variation in ligand enrichment by homology models based on the same template. Enrichment curves for 50 (A) D_2_R (based on D_3_R template) and (B) 5-HT_2A_R (based on 5-HT_2C_R template) homology models. Receiver operating characteristic (ROC) curves for databases of ligands and property-matched decoys ranked by molecular docking. The percentage of ligands identified and decoys found are shown on the y- and x-axis, respectively. The solid black line represents random enrichment of ligands.
(TIF)

S3 Fig Comparison of the ECL2 of the D_2_R and 5-HT_2A_R to template crystal structures. (A) Comparison of ECL2 of the D_2_R (green) to templates (β_1_AR, D_3_R, D_3_R, H_1_R, M_2_R, 5-HT_1B_R, 5-HT_2B_R, 5-HT_2C_R, A_2A_AR, CB1R, CXCR4, Rho; grey). (B) Comparison of the ECL2 of the 5-HT_2A_R (blue) to the 5-HT_2C_R template (grey). The receptor backbone is shown as cartoons. The conserved cysteine bridge formed by Cys^45.50^ is shown as sticks.
(TIF)

S4 Fig Relation between ligand enrichment and sequence identity. The median aLogAUC values of the D_2_R (A-B) and 5-HT_2A_R (C-D) homology models without ECL2 based on aminergic templates with different TM (A and C) or BS (B and D) sequence identities. The solid line represents a linear regression and R is Pearson’s correlation coefficient.
(TIF)

S5 Fig RMSD to the initial structure of the MD snapshots. TM backbone RMSDs of the D_2_R (A-C) and 5-HT_2A_R (D-F) MD snapshots to the initial homology model. The three trajectories of the Rho-based models (MD_Rho/Apo_, A and D) and models based on the most closely related template in apo (MD_Template/Apo_, B and E) and holo forms (MD_Template/Holo_, C and F) are shown.
(TIF)

S1 File D2R pairwise alignment. (ZIP)

S2 File 5-HT2AR pairwise alignment. (ZIP)

## References

[1] Lagerström MC, Schiöth HB. Structural diversity of G protein-coupled receptors and significance for drug discovery. Nat Rev Drug Discov. 2008;7: 339–357. 10.1038/nrd2518 18382464

[2] Hauser AS, Attwood MM, Rask-Andersen M, Schiöth HB, Gloriam DE. Trends in GPCR drug discovery: new agents, targets and indications. Nat Rev Drug Discov. 2017;16: 829–842. 10.1038/nrd.2017.178 29075003 PMC6882681

[3] Rodríguez D, Brea J, Loza MI, Carlsson J. Structure-Based Discovery of Selective Serotonin 5-HT1B Receptor Ligands. Structure. 2014;22: 1140–1151. 10.1016/j.str.2014.05.017 25043551

[4] De Graaf C, Kooistra AJ, Vischer HF, Katritch V, Kuijer M, Shiroishi M, et al Crystal structure-based virtual screening for fragment-like ligands of the human histamine H1 receptor. J Med Chem. 2011;54: 8195–8206. 10.1021/jm2011589 22007643 PMC3228891

[5] Kruse AC, Weiss DR, Rossi M, Hu J, Hu K, Eitel K, et al Muscarinic Receptors as Model Targets and Antitargets for Structure-Based Ligand Discovery. Mol Pharmacol. 2013;84: 528–540. 10.1124/mol.113.087551 23887926 PMC3781386

[6] Lane JR, Chubukov P, Liu W, Canals M, Cherezov V, Abagyan R, et al Structure-Based Ligand Discovery Targeting Orthosteric and Allosteric Pockets of Dopamine Receptors. Mol Pharmacol. 2013;84: 794–807. 10.1124/mol.113.088054 24021214 PMC3834142

[7] Kolb P, Rosenbaum DM, Irwin JJ, Fung JJ, Kobilka BK, Shoichet BK. Structure-based discovery of β2-adrenergic receptor ligands. Proc Natl Acad Sci U S A. 2009;106: 6843–6848. 10.1073/pnas.0812657106 19342484 PMC2672528

[8] Lyu J, Wang S, Balius TE, Singh I, Levit A, Moroz YS, et al Ultra-large library docking for discovering new chemotypes. Nature. 2019;566: 224–229. 10.1038/s41586-019-0917-9 30728502 PMC6383769

[9] Carlsson J, Yoo L, Gao Z-G, Irwin JJ, Shoichet BK, Jacobson KA. Structure-based discovery of A2A adenosine receptor ligands. J Med Chem. 2010;53: 3748–55. 10.1021/jm100240h 20405927 PMC2865168

[10] Ranganathan A, Heine P, Rudling A, Plückthun A, Kummer L, Carlsson J. Ligand Discovery for a Peptide-Binding GPCR by Structure-Based Screening of Fragment- and Lead-Like Chemical Libraries. ACS Chem Biol. 2017;12: 735–745. 10.1021/acschembio.6b00646 28032980

[11] Manglik A, Lin H, Aryal DK, McCorvy JD, Dengler D, Corder G, et al Structure-based discovery of opioid analgesics with reduced side effects. Nature. 2016;537: 185–190. 10.1038/nature19112 27533032 PMC5161585

[12] Mysinger MM, Weiss DR, Ziarek JJ, Gravel S, Doak AK, Karpiak J, et al Structure-based ligand discovery for the protein-protein interface of chemokine receptor CXCR4. Proc Natl Acad Sci. 2012;109: 5517–5522. 10.1073/pnas.1120431109 22431600 PMC3325704

[13] Pándy-Szekeres G, Munk C, Tsonkov TM, Mordalski S, Harpsøe K, Hauser AS, et al GPCRdb in 2018: Adding GPCR structure models and ligands. Nucleic Acids Res. 2018;46: D440–D446. 10.1093/nar/gkx1109 29155946 PMC5753179

[14] Baker D, Sali A. Protein structure prediction and structural genomics. Science. 2001;294: 93–96. 10.1126/science.1065659 11588250

[15] Michino M, Abola E, Brooks CL, Scott Dixon J, Moult J, Stevens RC, et al Community-wide assessment of GPCR structure modelling and ligand docking: GPCR Dock 2008. Nat Rev Drug Discov. 2009;8: 455–463. 10.1038/nrd2877 19461661 PMC2728591

[16] Kufareva I, Rueda M, Katritch V, Stevens RC, Abagyan R, Yoshikawa Y, et al Status of GPCR modeling and docking as reflected by community-wide GPCR Dock 2010 assessment. Structure. 2011;19: 1108–1126. 10.1016/j.str.2011.05.012 21827947 PMC3154726

[17] Kufareva I, Katritch V, Participants of GPCR Dock 2013, Stevens RC, Abagyan R. Advances in GPCR modeling evaluated by the GPCR dock 2013 assessment: Meeting new challenges. Structure. 2014;22: 1120–1139. 10.1016/j.str.2014.06.012 25066135 PMC4126895

[18] Rodríguez D, Ranganathan A, Carlsson J. Strategies for improved modeling of GPCR-drug complexes: Blind predictions of serotonin receptors bound to ergotamine. J Chem Inf Model. 2014;54: 2004–2021. 10.1021/ci5002235 25030302

[19] Katritch V, Kufareva I, Abagyan R. Structure based prediction of subtype-selectivity for adenosine receptor antagonists. Neuropharmacology. 2011;60: 108–115. 10.1016/j.neuropharm.2010.07.009 20637786 PMC2980563

[20] Phatak SS, Gatica EA, Cavasotto CN. Ligand-steered modeling and docking: A benchmarking study in class A G-protein-coupled receptors. J Chem Inf Model. 2010;50: 2119–28. 10.1021/ci100285f 21080692

[21] Kołaczkowski M, Bucki A, Feder M, Pawłowski M. Ligand-optimized homology models of D1 and D2 dopamine receptors: application for virtual screening. J Chem Inf Model. 2013;53: 638–48. 10.1021/ci300413h 23398329

[22] McRobb FM, Capuano B, Crosby IT, Chalmers DK, Yuriev E. Homology modeling and docking evaluation of aminergic G protein-coupled receptors. J Chem Inf Model. 2010;50: 626–37. 10.1021/ci900444q 20187660

[23] Sirci F, Istyastono EP, Vischer HF, Kooistra AJ, Nijmeijer S, Kuijer M, et al Virtual fragment screening: discovery of histamine H3 receptor ligands using ligand-based and protein-based molecular fingerprints. J Chem Inf Model. 2012;52: 3308–24. 10.1021/ci3004094 23140085

[24] Costanzi S, Cohen A, Danfora A, Dolatmoradi M. Influence of the Structural Accuracy of Homology Models on Their Applicability to Docking-Based Virtual Screening: The β2 Adrenergic Receptor as a Case Study. J Chem Inf Model. 2019;59: 3177–3190. 10.1021/acs.jcim.9b00380 31257873 PMC6800073

[25] Lam VM, Rodríguez D, Zhang T, Koh EJ, Carlsson J, Salahpour A. Discovery of trace amine-associated receptor 1 ligands by molecular docking screening against a homology model. Medchemcomm. 2015;6: 2216–2223. 10.1039/c5md00400d

[26] Ranganathan A, Stoddart LA, Hill SJ, Carlsson J. Fragment-Based Discovery of Subtype-Selective Adenosine Receptor Ligands from Homology Models. J Med Chem. 2015;58: 9578–9590. 10.1021/acs.jmedchem.5b01120 26592528

[27] Huang X-P, Karpiak J, Kroeze WK, Zhu H, Chen X, Moy SS, et al Allosteric ligands for the pharmacologically dark receptors GPR68 and GPR65. Nature. 2015;527: 477–83. 10.1038/nature15699 26550826 PMC4796946

[28] Weiss DR, Karpiak J, Huang X-P, Sassano MF, Lyu J, Roth BL, et al Selectivity Challenges in Docking Screens for GPCR Targets and Antitargets. J Med Chem. 2018;61: 6830–6845. 10.1021/acs.jmedchem.8b00718 29990431 PMC6105036

[29] Carlsson J, Coleman RG, Setola V, Irwin JJ, Fan H, Schlessinger A, et al Ligand discovery from a dopamine D3 receptor homology model and crystal structure. Nat Chem Biol. 2011;7: 769–778. 10.1038/nchembio.662 21926995 PMC3197762

[30] Istyastono EP, Kooistra AJ, Vischer HF, Kuijer M, Roumen L, Nijmeijer S, et al Structure-based virtual screening for fragment-like ligands of the G protein-coupled histamine H4 receptor. Medchemcomm. 2015;6: 1003–1017. 10.1039/c5md00022j

[31] Vass M, Schmidt É, Horti F, Keseru GM. Virtual fragment screening on GPCRs: A case study on dopamine D3 and histamine H4 receptors. Eur J Med Chem. 2014;77: 38–46. 10.1016/j.ejmech.2014.02.034 24607587

[32] Fan H, Irwin JJ, Webb BM, Klebe G, Shoichet BK, Sali A. Molecular Docking Screens Using Comparative Models of Proteins. J Chem Inf Model. 2009;49: 2512–2527. 10.1021/ci9003706 19845314 PMC2790034

[33] Rataj K, Witek J, Mordalski S, Kosciolek T, Bojarski AJ. Impact of template choice on homology model efficiency in virtual screening. J Chem Inf Model. 2014;54: 1661–1668. 10.1021/ci500001f 24813470

[34] Lim VJY, Du W, Chen YZ, Fan H. A benchmarking study on virtual ligand screening against homology models of human GPCRs. Proteins Struct Funct Bioinforma. 2018;86: 978–989. 30051928 10.1002/prot.25533

[35] Tarcsay A, Paragi G, Vass M, Jójárt B, Bogár F, Keserű GM. The impact of molecular dynamics sampling on the performance of virtual screening against GPCRs. J Chem Inf Model. 2013;53: 2990–9. 10.1021/ci400087b 24116387

[36] Roth BL, Sheffler DJ, Kroeze WK. Magic shotguns versus magic bullets: selectively non-selective drugs for mood disorders and schizophrenia. Nat Rev Drug Discov. 2004;3: 353–359. 10.1038/nrd1346 15060530

[37] Warne T, Serrano-Vega MJ, Baker JG, Moukhametzianov R, Edwards PC, Henderson R, et al Structure of a β1-adrenergic G-protein-coupled receptor. Nature. 2008;454: 486–491. 10.1038/nature07101 18594507 PMC2923055

[38] Cherezov V, Rosenbaum DM, Hanson MA, Rasmussen SGF, Foon ST, Kobilka TS, et al High-resolution crystal structure of an engineered human β2-adrenergic G protein-coupled receptor. Science. 2007;318: 1258–1265. 10.1126/science.1150577 17962520 PMC2583103

[39] Chien EYT, Liu W, Zhao Q, Katritch V, Han GW, Hanson MA, et al Structure of the human dopamine D3 receptor in complex with a D2/D3 selective antagonist. Science. 2010;330: 1091–1095. 10.1126/science.1197410 21097933 PMC3058422

[40] Wang S, Wacker D, Levit A, Che T, Betz RM, McCorvy JD, et al D4 dopamine receptor high-resolution structures enable the discovery of selective agonists. Science. 2017;358: 381–386. 10.1126/science.aan5468 29051383 PMC5856174

[41] Shimamura T, Shiroishi M, Weyand S, Tsujimoto H, Winter G, Katritch V, et al Structure of the human histamine H1 receptor complex with doxepin. Nature. 2011;475: 65–72. 10.1038/nature10236 21697825 PMC3131495

[42] Wang C, Jiang Y, Ma J, Wu H, Wacker D, Katritch V, et al Structural Basis for Molecular Recognition at Serotonin Receptors. Science. 2013;340: 610–614. 10.1126/science.1232807 23519210 PMC3644373

[43] Wacker D, Wang C, Katritch V, Han GW, Huang XP, Vardy E, et al Structural features for functional selectivity at serotonin receptors. Science. 2013;340: 615–619. 10.1126/science.1232808 23519215 PMC3644390

[44] Peng Y, McCorvy JD, Harpsøe K, Lansu K, Yuan S, Popov P, et al 5-HT2C Receptor Structures Reveal the Structural Basis of GPCR Polypharmacology. Cell. 2018;172: 719–730.e14. 10.1016/j.cell.2018.01.001 29398112 PMC6309861

[45] Thal DM, Sun B, Feng D, Nawaratne V, Leach K, Felder CC, et al Crystal structures of the M1 and M4 muscarinic acetylcholine receptors. Nature. 2016;531: 335–40. 10.1038/nature17188 26958838 PMC4915387

[46] Haga K, Kruse AC, Asada H, Yurugi-Kobayashi T, Shiroishi M, Zhang C, et al Structure of the human M2 muscarinic acetylcholine receptor bound to an antagonist. Nature. 2012;482: 547–51. 10.1038/nature10753 22278061 PMC3345277

[47] Kruse AC, Hu J, Pan AC, Arlow DH, Rosenbaum DM, Rosemond E, et al Structure and dynamics of the M3 muscarinic acetylcholine receptor. Nature. 2012;482: 552–556. 10.1038/nature10867 22358844 PMC3529910

[48] Palczewski K, Kumasaka T, Hori T, Behnke CA, Motoshima H, Fox BA, et al Crystal Structure of Rhodopsin: A G Protein-Coupled Receptor. Science. 2000;289: 739–745. 10926528 10.1126/science.289.5480.739

[49] Wu B, Chien EYT, Mol CD, Fenalti G, Liu W, Katritch V, et al Structures of the CXCR4 chemokine GPCR with small-molecule and cyclic peptide antagonists. Science. 2010;330: 1066–1071. 10.1126/science.1194396 20929726 PMC3074590

[50] Liu W, Chun E, Thompson AA, Chubukov P, Xu F, Katritch V, et al Structural basis for allosteric regulation of GPCRs by sodium ions. Science. 2012;337: 232–236. 10.1126/science.1219218 22798613 PMC3399762

[51] Shao Z, Yin J, Chapman K, Grzemska M, Clark L, Wang J, et al High-resolution crystal structure of the human CB1 cannabinoid receptor. Nature. 2016;540: 602–606. 10.1038/nature20613 27851727 PMC5433929

[52] Michino M, Beuming T, Donthamsetti P, Newman AH, Javitch JA, Shi L. What can crystal structures of aminergic receptors tell us about designing subtype-selective ligands? Pharmacol Rev. 2015;67: 198–213. 10.1124/pr.114.009944 25527701 PMC4279073

[53] Šali A, Blundell TL. Comparative protein modelling by satisfaction of spatial restraints. J Mol Biol. 1993;234: 779–815. 10.1006/jmbi.1993.1626 8254673

[54] Shen M-Y, Sali A. Statistical potential for assessment and prediction of protein structures. Protein Sci. 2006;15: 2507–2524. 10.1110/ps.062416606 17075131 PMC2242414

[55] Wang S, Che T, Levit A, Shoichet BK, Wacker D, Roth BL. Structure of the D2 dopamine receptor bound to the atypical antipsychotic drug risperidone. Nature. 2018;555: 269–273. 10.1038/nature25758 29466326 PMC5843546

[56] Kimura KT, Asada H, Inoue A, Kadji FMN, Im D, Mori C, et al Structures of the 5-HT2A receptor in complex with the antipsychotics risperidone and zotepine. Nat Struct Mol Biol. 2019;26: 121–128. 10.1038/s41594-018-0180-z 30723326

[57] Mysinger MM, Shoichet BK. Rapid context-dependent ligand desolvation in molecular docking. J Chem Inf Model. 2010;50: 1561–1573. 10.1021/ci100214a 20735049

[58] Lorber DM, Shoichet BK. Hierarchical docking of databases of multiple ligand conformations. Curr Top Med Chem. 2005;5: 739–49. 10.2174/1568026054637683 16101414 PMC1364474

[59] Mysinger MM, Carchia M, Irwin JJ, Shoichet BK. Directory of useful decoys, enhanced (DUD-E): Better ligands and decoys for better benchmarking. J Med Chem. 2012;55: 6582–6594. 10.1021/jm300687e 22716043 PMC3405771

[60] Ballesteros JA, Weinstein H. Integrated methods for the construction of three-dimensional models and computational probing of structure-function relations in G protein-coupled receptors. Methods Neurosci. 1995;25: 366–428. 10.1016/S1043-9471(05)80049-7

[61] Vass M, Podlewska S, De Esch IJP, Bojarski AJ, Leurs R, Kooistra AJ, et al Aminergic GPCR-Ligand Interactions: A Chemical and Structural Map of Receptor Mutation Data. J Med Chem. 2019;62: 3784–3839. 10.1021/acs.jmedchem.8b00836 30351004

[62] Kairys V, Fernandes MX, Gilson MK. Screening drug-like compounds by docking to homology models: A systematic study. J Chem Inf Model. 2006;46: 365–379. 10.1021/ci050238c 16426071

[63] de Graaf C, Foata N, Engkvist O, Rognan D. Molecular modeling of the second extracellular loop of G-protein coupled receptors and its implication on structure-based virtual screening. Proteins. 2008;71: 599–620. 10.1002/prot.21724 17972285

[64] Muntasir HA, Bhuiyan MA, Ishiguro M, Ozaki M, Nagatomo T. Inverse agonist activity of sarpogrelate, a selective 5-HT2A -receptor antagonist, at the constitutively active human 5-HT2A receptor. J Pharmacol Sci. 2006;102: 189–195. 10.1254/jphs.fp0060610 17031071

[65] Cao J, Slack RD, Bakare OM, Burzynski C, Rais R, Slusher BS, et al Novel and High Affinity 2-[(Diphenylmethyl)sulfinyl]acetamide (Modafinil) Analogues as Atypical Dopamine Transporter Inhibitors. J Med Chem. 2016;59: 10676–10691. 10.1021/acs.jmedchem.6b01373 27933960 PMC5161041

[66] Weis WI, Kobilka BK. The Molecular Basis of G Protein-Coupled Receptor Activation. Annu Rev Biochem. 2018;87: 897–919. 10.1146/annurev-biochem-060614-033910 29925258 PMC6535337

[67] Weiss DR, Ahn S, Sassano MF, Kleist A, Zhu X, Strachan R, et al Conformation guides molecular efficacy in docking screens of activated β-2 adrenergic G protein coupled receptor. ACS Chem Biol. 2013;8: 1018–1026. 10.1021/cb400103f 23485065 PMC3658555

[68] Männel B, Jaiteh M, Zeifman A, Randakova A, Möller D, Hübner H, et al Structure-Guided Screening for Functionally Selective D2 Dopamine Receptor Ligands from a Virtual Chemical Library. ACS Chem Biol. 2017;12: 2652–2661. 10.1021/acschembio.7b00493 28846380

[69] Totrov M, Abagyan R. Flexible ligand docking to multiple receptor conformations: a practical alternative. Curr Opin Struct Biol. 2008;18: 178–84. 10.1016/j.sbi.2008.01.004 18302984 PMC2396190

[70] Raval A, Piana S, Eastwood MP, Dror RO, Shaw DE. Refinement of protein structure homology models via long, all-atom molecular dynamics simulations. Proteins Struct Funct Bioinforma. 2012;80: 2071–2079. 22513870 10.1002/prot.24098

[71] Heo L, Feig M. What makes it difficult to refine protein models further via molecular dynamics simulations? Proteins. 2018;86 Suppl 1: 177–188. 10.1002/prot.25393 28975670 PMC5820117

[72] The UniProt Consortium. UniProt: A worldwide hub of protein knowledge. Nucleic Acids Res. 2019;47: D506–D515. 10.1093/nar/gky1049 30395287 PMC6323992

[73] Katoh K, Standley DM. MAFFT multiple sequence alignment software version 7: Improvements in performance and usability. Mol Biol Evol. 2013;30: 772–780. 10.1093/molbev/mst010 23329690 PMC3603318

[74] Eddy SR. Profile hidden Markov models. Bioinformatics. 1998;14: 755–763. 10.1093/bioinformatics/14.9.755 9918945

[75] Schrödinger LLC. The PyMOL Molecular Graphics System, Version~2.0. 2017.

[76] Bento AP, Gaulton A, Hersey A, Bellis LJ, Chambers J, Davies M, et al The ChEMBL bioactivity database: An update. Nucleic Acids Res. 2014;42: D1083–D1090. 10.1093/nar/gkt1031 24214965 PMC3965067

[77] Sterling T, Irwin JJ. ZINC 15—Ligand Discovery for Everyone. J Chem Inf Model. 2015;55: 2324–37. 10.1021/acs.jcim.5b00559 26479676 PMC4658288

[78] Irwin JJ, Shoichet BK. ZINC − A Free Database of Commercially Available Compounds for Virtual Screening. J Chem Inf Model. 2005;45: 177–182. 10.1021/ci049714 15667143 PMC1360656

[79] Olsson MHM, Søndergaard CR, Rostkowski M, Jensen JH. PROPKA3: Consistent Treatment of Internal and Surface Residues in Empirical pKa Predictions. J Chem Theory Comput. 2011;7: 525–37. 10.1021/ct100578z 26596171

[80] Lomize MA, Pogozheva ID, Joo H, Mosberg HI, Lomize AL. OPM database and PPM web server: Resources for positioning of proteins in membranes. Nucleic Acids Res. 2012;40: D370–D376. 10.1093/nar/gkr703 21890895 PMC3245162

[81] Russell RB, Barton GJ. Multiple protein sequence alignment from tertiary structure comparison: Assignment of global and residue confidence levels. Proteins Struct Funct Bioinforma. 1992;14: 309–323. 10.1002/prot.340140216 1409577

[82] Doerr S, Harvey MJ, Noé F, De Fabritiis G. HTMD: High-Throughput Molecular Dynamics for Molecular Discovery. J Chem Theory Comput. 2016;12: 1845–1852. 10.1021/acs.jctc.6b00049 26949976

[83] Huang J, Rauscher S, Nawrocki G, Ran T, Feig M, de Groot BL, et al CHARMM36m: an improved force field for folded and intrinsically disordered proteins. Nat Methods. 2017;14: 71–73. 10.1038/nmeth.4067 27819658 PMC5199616

[84] Andersen HC. Rattle: A “velocity” version of the shake algorithm for molecular dynamics calculations. J Comput Phys. 1983;52: 24–34. 10.1016/0021-9991(83)90014-1

[85] Vanommeslaeghe K, Hatcher E, Acharya C, Kundu S, Zhong S, Shim J, et al CHARMM general force field: A force field for drug-like molecules compatible with the CHARMM all-atom additive biological force fields. J Comput Chem. 2010;31: 671–90. 10.1002/jcc.21367 19575467 PMC2888302

[86] Yu W, He X, Vanommeslaeghe K, MacKerell AD. Extension of the CHARMM General Force Field to sulfonyl-containing compounds and its utility in biomolecular simulations. J Comput Chem. 2012;33: 2451–68. 10.1002/jcc.23067 22821581 PMC3477297

[87] Harvey MJ, Giupponi G, De Fabritiis G. ACEMD: Accelerating biomolecular dynamics in the microsecond time scale. J Chem Theory Comput. 2009;5: 1632–1639. 10.1021/ct9000685 26609855

[88] Feenstra KA, Hess B, Berendsen HJC. Improving efficiency of large time-scale molecular dynamics simulations of hydrogen-rich systems. J Comput Chem. 1999;20: 786–798. 10.1002/(SICI)1096-987X(199906)20:8<786::AID-JCC5>3.0.CO;2-B 35619462

[89] Essmann U, Perera L, Berkowitz ML, Darden T, Lee H, Pedersen LG. A smooth particle mesh Ewald method. J Chem Phys. 1995;103: 8577–8593. 10.1063/1.470117

[90] Michaud-Agrawal N, Denning EJ, Woolf TB, Beckstein O. MDAnalysis: a toolkit for the analysis of molecular dynamics simulations. J Comput Chem. 2011;32: 2319–27. 10.1002/jcc.21787 21500218 PMC3144279

[91] Gowers R, Linke M, Barnoud J, Reddy T, Melo M, Seyler S, et al MDAnalysis: A Python Package for the Rapid Analysis of Molecular Dynamics Simulations. Proceedings of the 15th Python in Science Conference. 2016 pp. 98–105. 10.25080/majora-629e541a-00e

[92] McGibbon RT, Beauchamp KA, Harrigan MP, Klein C, Swails JM, Hernández CX, et al MDTraj: A Modern Open Library for the Analysis of Molecular Dynamics Trajectories. Biophys J. 2015;109: 1528–32. 10.1016/j.bpj.2015.08.015 26488642 PMC4623899

[93] Tubiana T, Carvaillo J-C, Boulard Y, Bressanelli S. TTClust: A Versatile Molecular Simulation Trajectory Clustering Program with Graphical Summaries. J Chem Inf Model. 2018;58: 2178–2182. 10.1021/acs.jcim.8b00512 30351057

