# Supplementary material for: Performance of virtual screening against GPCR homology models: Impact of template selection and treatment of binding site plasticity
Source: PLoS Comput Biol. 2020 Mar 13;16(3):e1007680. doi: 10.1371/journal.pcbi.1007680 (PMC7135368; doi:10.1371/journal.pcbi.1007680)
Supplement: S1 Table — (PDF) [file pcbi.1007680.s001.pdf]

S1 Table. Definition of the TM helix region using Ballesteros-Weinstein numbering [60].

TM1	TM2	TM3	TM4	TM5	TM6	TM7
1.33 – 1.57	2.40 – 2.67	3.22 – 3.55	4.41 – 4.63	5.37 – 5.68	6.29– 6.62	7.30 – 7.57
