# Supplementary material for: Performance of virtual screening against GPCR homology models: Impact of template selection and treatment of binding site plasticity
Source: PLoS Comput Biol. 2020 Mar 13;16(3):e1007680. doi: 10.1371/journal.pcbi.1007680 (PMC7135368; doi:10.1371/journal.pcbi.1007680)
Supplement: S2 Table — Statistics are based on 50 homology models per template. (PDF) [file pcbi.1007680.s002.pdf]

S2 Table. Average pairwise RMSDs for the binding site side chains of the D_2R and 5-$HT_{2A}R$ homology models. Statistics are based on 50 homology models per template.

Template	Pairwise RMSD of binding site side chains (Å)[a]	
	D_2R	5-$HT_{2A}R$
β_1AR	1.1 ± 0.1	1.1 ± 0.1
β_2AR	1.2 ± 0.1	1.1 ± 0.2
D_3R	1.0 ± 0.1	1.2 ± 0.1
D_4R	1.2 ± 0.2	1.6 ± 0.4
H_1R	1.4 ± 0.2	1.3 ± 0.2
M_1R	1.5 ± 0.2	1.5 ± 0.2
M_2R	1.5 ± 0.2	1.5 ± 0.2
M_3R	1.4 ± 0.2	1.3 ± 0.2
M_4R	1.4 ± 0.2	1.4 ± 0.2
5-$HT_{1B}R$	1.3 ± 0.2	1.3 ± 0.2
5-$HT_{2B}R$	1.4 ± 0.2	0.9 ± 0.1
5-$HT_{2C}R$	1.4 ± 0.3	0.9 ± 0.1
Rho	1.7 ± 0.3	1.8 ± 0.3
CXCR4	1.7 ± 0.2	1.6 ± 0.2
$A_{2A}AR$	1.9 ± 0.3	2.0 ± 0.3
CB1R	2.0 ± 0.3	2.0 ± 0.3

[a]Average RMSD ± standard deviation.
