# Supplementary material for: Performance of virtual screening against GPCR homology models: Impact of template selection and treatment of binding site plasticity
Source: PLoS Comput Biol. 2020 Mar 13;16(3):e1007680. doi: 10.1371/journal.pcbi.1007680 (PMC7135368; doi:10.1371/journal.pcbi.1007680)
Supplement: S3 Table — Statistics are based on 50 homology models per template. (PDF) [file pcbi.1007680.s003.pdf]

S3 Table. Average RMSDs of D_2R and 5-$HT_{2A}R$ homology models to the crystal structures. Statistics are based on 50 homology models per template.

Template	RMSD (Å)					
	D_2R[a]			5-$HT_{2A}R$[b]		
	BSSC[c]	BSBB[d]	TMBB[e]	BSSC[c]	BSBB[d]	TMBB[e]
**β_1AR**	2.4 ± 0.1	1.7 ± 0.1	1.6 ± 0.0	1.6 ± 0.1	1.2 ± 0.0	1.7 ± 0.1
**β_2AR**	2.4 ± 0.1	1.7 ± 0.1	1.7 ± 0.0	1.7 ± 0.1	1.3 ± 0.1	1.7 ± 0.1
**D_3R**	3.0 ± 0.1	1.8 ± 0.0	1.3 ± 0.0	2.6 ± 0.1	1.1 ± 0.1	1.6 ± 0.1
**D_4R**	2.9 ± 0.1	1.9 ± 0.0	1.7 ± 0.0	2.1 ± 0.3	1.1 ± 0.0	1.5 ± 0.1
**H_1R**	2.8 ± 0.1	1.8 ± 0.1	1.8 ± 0.0	2.0 ± 0.1	1.4 ± 0.0	1.8 ± 0.1
**M_1R**	3.0 ± 0.1	1.6 ± 0.1	2.1 ± 0.0	2.6 ± 0.1	1.5 ± 0.0	1.9 ± 0.1
**M_2R**	3.0 ± 0.2	1.6 ± 0.1	1.9 ± 0.0	2.4 ± 0.2	1.4 ± 0.1	1.9 ± 0.1
**M_3R**	3.2 ± 0.1	1.8 ± 0.1	2.1 ± 0.0	2.3 ± 0.1	1.3 ± 0.1	1.9 ± 0.1
**M_4R**	3.1 ± 0.2	1.7 ± 0.1	2.0 ± 0.1	2.4 ± 0.1	1.4 ± 0.0	1.8 ± 0.1
**5-$HT_{1B}R$**	3.2 ± 0.1	2.2 ± 0.1	2.2 ± 0.1	2.2 ± 0.1	1.6 ± 0.1	2.3 ± 0.1
**5-$HT_{2B}R$**	2.8 ± 0.1	1.9 ± 0.1	2.0 ± 0.0	1.7 ± 0.1	1.0 ± 0.0	1.7 ± 0.0
**5-$HT_{2C}R$**	2.6 ± 0.1	1.5 ± 0.1	1.6 ± 0.0	1.5 ± 0.1	1.1 ± 0.0	1.5 ± 0.0
**Rho**	3.1 ± 0.1	2.5 ± 0.1	2.2 ± 0.0	2.8 ± 0.2	1.9 ± 0.0	2.1 ± 0.0
**CXCR4**	3.1 ± 0.1	1.7 ± 0.1	2.2 ± 0.0	2.8 ± 0.1	1.5 ± 0.1	2.3 ± 0.0
**$A_{2A}AR$**	3.6 ± 0.2	2.1 ± 0.1	1.8 ± 0.0	4.5 ± 0.2	2.2 ± 0.0	1.8 ± 0.0
**CB1R**	4.0 ± 0.2	2.8 ± 0.1	2.3 ± 0.1	3.6 ± 0.2	2.4 ± 0.2	2.0 ± 0.1

[a]Average RMSD ± standard deviation to the D_2R crystal structure (PDB code: 6CM4).
[b]Average RMSD ± standard deviation to the 5-$HT_{2A}R$ crystal structure (PDB code: 6A94, chain B).
[c]Binding site side chains (BSSC).
[d]Binding site backbone (BSBB).
[e]Transmembrane backbone (TMBB).
