# Supplementary material for: Performance of virtual screening against GPCR homology models: Impact of template selection and treatment of binding site plasticity
Source: PLoS Comput Biol. 2020 Mar 13;16(3):e1007680. doi: 10.1371/journal.pcbi.1007680 (PMC7135368; doi:10.1371/journal.pcbi.1007680)
Supplement: S4 Table — Statistics are based on 50 homology models per template. (PDF) [file pcbi.1007680.s004.pdf]

S4 Table. Ligand enrichment (aLogAUC) by D_2R and $5\text{-}HT_{2A}R$ homology models based on different templates. Statistics are based on 50 homology models per template.

**Template**	**aLogAUC**					
	**D_2R**			**$5\text{-}HT_{2A}R$**		
	**Median**	**Max**	**Ensemble[a]**	**Median**	**Max**	**Ensemble[a]**
**β_1AR**	20.4	24.1	21.1	21.9	28.3	21.5
**β_2AR**	19.2	24.3	23.9	24.0	28.2	21.9
**D_3R**	16.7	22.5	19.6	16.0	28.8	23.1
**D_4R**	9.0	17.8	18.1	12.9	22.3	15.7
**H_1R**	17.8	24.1	20.3	20.4	28.3	16.6
**M_1R**	12.0	17.9	16.5	11.6	16.7	11.7
**M_2R**	13.6	17.8	16.9	10.8	16.8	9.4
**M_3R**	13.2	21.5	16.0	11.6	15.3	10.2
**M_4R**	11.9	16.4	18.1	11.5	15.2	12.5
**$5\text{-}HT_{1B}R$**	15.3	20.2	17.7	20.4	28.6	21.7
**$5\text{-}HT_{2B}R$**	18.1	23.7	22.6	23.9	30.4	26.1
**$5\text{-}HT_{2C}R$**	18.9	26.1	26.0	23.9	30.3	26.1
**all aminergic**	-	-	21.1	-	-	23.8
**Rho**	–6.2	–2.3	–4.4	4.4	14.4	16.1
**CXCR4**	18.8	24.4	16.7	13.8	21.4	21.5
**$A_{2A}AR$**	7.4	19.7	11.6	11.3	19.6	14.7
**CB1R**	15.3	21.9	17.4	15.6	23.1	14.9
**D_3R/D_2R_{ECL2}[b]**	23.6	26.5	25.1	-	-	-

[a]The ensemble enrichment was calculated by identifying the best docking score of each docked compound among multiple homology models, leading to a single aLogAUC value for the set. The ensemble enrichment was calculated for each template (50 models) and all aminergic templates (600 models).

[b]D_2R homology models based on the D_3R with the structure of ECL2 extracted from the D_2R crystal structure.
