# Supplementary material for: Performance of virtual screening against GPCR homology models: Impact of template selection and treatment of binding site plasticity
Source: PLoS Comput Biol. 2020 Mar 13;16(3):e1007680. doi: 10.1371/journal.pcbi.1007680 (PMC7135368; doi:10.1371/journal.pcbi.1007680)
Supplement: S5 Table — (PDF) [file pcbi.1007680.s005.pdf]

**S5 Table.** Ligand enrichment (aLogAUC) by the $D_2R$ and 5-$HT_{2A}R$ crystal structures.

| **Target:** | **$D_2R$** | **5-$HT_{2A}R$[a]** | |
|---|---|---|---|
| **PDB code** | 6CM4 | 6A93 | 6A94 |
| **aLogAUC** | 26.6 | 23.7/23.4 | 22.5/26.1 |

[a]Chain A/Chain B of crystal structure.
