# Supplementary material for: Performance of virtual screening against GPCR homology models: Impact of template selection and treatment of binding site plasticity
Source: PLoS Comput Biol. 2020 Mar 13;16(3):e1007680. doi: 10.1371/journal.pcbi.1007680 (PMC7135368; doi:10.1371/journal.pcbi.1007680)
Supplement: S6 Table — (PDF) [file pcbi.1007680.s006.pdf]

S6 Table. Ligand enrichment (aLogAUC) by crystal structure templates.

Template	aLogAUC	
	D_2R	$5\text{-}HT_{2A}R$
β_1AR	20.4	24.0
β_2AR	18.9	25.4
D_3R	13.8	6.4
D_4R	16.9	12.7
H_1R	–0.9	9.3
M_1R	–3.9	7.7
M_2R	1.7	10.5
M_3R	–3.4	9.3
M_4R	–5.5	4.5
$5\text{-}HT_{1B}R$	21.8	24.0
$5\text{-}HT_{2B}R$	16.6	14.7
$5\text{-}HT_{2C}R$	25.9	27.6
Rho	0.4	6.4
CXCR4	14.4	9.4
$A_{2A}AR$	2.9	6.3
CB1R	1.1	3.0
