# Supplementary material for: Performance of virtual screening against GPCR homology models: Impact of template selection and treatment of binding site plasticity
Source: PLoS Comput Biol. 2020 Mar 13;16(3):e1007680. doi: 10.1371/journal.pcbi.1007680 (PMC7135368; doi:10.1371/journal.pcbi.1007680)
Supplement: S7 Table — Statistics are based on 50 homology models per template. (PDF) [file pcbi.1007680.s007.pdf]

S7 Table. Ligand enrichment (aLogAUC) by D_2R and 5-$HT_{2A}R$ homology models without ECL2. Statistics are based on 50 homology models per template.

Template	aLogAUC					
	D_2R			5-$HT_{2A}R$		
	Median	Max	Ensemble[a]	Median	Max	Ensemble[a]
$β_1AR$	23.4	27.8	25.7	23.8	29.5	20.1
$β_2AR$	21.3	27.6	25.7	25.7	30.0	23.1
D_3R	24.9	30.7	27.0	21.9	29.4	22.2
D_4R	18.2	26.3	22.8	18.5	28.2	17.9
H_1R	19.2	23.7	21.6	21.3	29.7	24.2
M_1R	13.1	18.4	15.1	10.8	17.8	11.7
M_2R	14.0	19.3	16.2	10.1	16.8	8.9
M_3R	13.6	23.0	14.6	11.1	15.1	8.8
M_4R	14.2	19.8	17.8	11.3	16.3	12.6
5-$HT_{1B}R$	20.2	24.3	21.9	22.2	29.7	21.0
5-$HT_{2B}R$	20.0	26.0	24.1	25.6	32.7	26.1
5-$HT_{2C}R$	23.6	27.9	25.4	25.8	31.1	29.4
all aminergic	-	-	23.1	-	-	18.2
Rho	7.3	16.8	14.2	12.6	21.9	18.5
CXCR4	19.6	26.6	18.4	15.0	20.3	12.7
$A_{2A}AR$	11.0	17.9	11.2	15.8	24.4	18.3
CB1R	15.3	21.6	17.2	15.7	22.3	16.1

[a]The ensemble enrichment was calculated by identifying the best docking score of each docked compound among multiple homology models, leading to a single aLogAUC value for the set. The ensemble enrichment was calculated for each template (50 models) and all aminergic templates (600 models).
