# Supplementary material for: Performance of virtual screening against GPCR homology models: Impact of template selection and treatment of binding site plasticity
Source: PLoS Comput Biol. 2020 Mar 13;16(3):e1007680. doi: 10.1371/journal.pcbi.1007680 (PMC7135368; doi:10.1371/journal.pcbi.1007680)
Supplement: S8 Table — (PDF) [file pcbi.1007680.s008.pdf]

S8 Table. Smiles of D_2R ligands similar to the co-crystallized ligands of three templates (doxepin-, eticlopride-, piperidine/piperazine-like ligands) from the ChEMBL database [76].

SMILE	ChEMBL ID	Chemotype
CN(C)CCCN1c2ccccc2CCc3c1cccc3	CHEMBL11	Doxepin
CN1CCN(CC1)C2=Nc3cc(ccc3Nc4c2cccc4)Cl	CHEMBL42	Doxepin
CN1CCC(=C2c3ccccc3C=Cc4c2cccc4)CC1	CHEMBL516	Doxepin
CN(C)CCC=C1c2ccccc2CCc3c1cccc3	CHEMBL629	Doxepin
CN1CCN(CC1)C2=Nc3ccccc3Oc4c2cc(cc4)Cl	CHEMBL831	Doxepin
CN1CCC(CC1)C2=Cc3cc(ccc3Cc4c2cccc4)Cl	CHEMBL7430	Doxepin
CN1CCN(CC1)C2=Cc3ccccc3Cc4c2cc(cc4)Cl	CHEMBL7617	Doxepin
CN1CCN(CC1)C2=Nc3cc(ccc3Oc4c2cccc4)Cl	CHEMBL7828	Doxepin
CN(C)C1CCC2(C=C1)c3ccccc3CCc4c2cc(cc4)Cl	CHEMBL45491	Doxepin
CN1CCN(CC1)C2Cc3ccccc3Sc4c2cc(cc4)Cl	CHEMBL64249	Doxepin
CN1CCN(CC1)C2=Cc3cc(ccc3Oc4c2cccc4)Cl	CHEMBL88464	Doxepin
CC(=C1c2ccccc2C=C(c3c1cccc3)N4CCN(CC4)C)C	CHEMBL88870	Doxepin
CN1CCN(CC1)C2=Cc3ccccc3Oc4c2cccc4	CHEMBL89970	Doxepin
CC1c2ccccc2C=C(c3c1cccc3)C4=CCN(CC4)C	CHEMBL90654	Doxepin
CN1CCN(CC1)C2=Cc3ccccc3Oc4c2cc(cc4)Cl	CHEMBL90882	Doxepin
CC=C1c2ccccc2C=C(c3c1cccc3)N4CCN(CC4)C	CHEMBL90977	Doxepin
CN1CCN(CC1)C2=Cc3ccccc3C(=C)c4c2cccc4	CHEMBL91310	Doxepin
CC1c2ccccc2C=C(c3c1cccc3)C4=CCN(CC4)C	CHEMBL91350	Doxepin
CC(=C1c2ccccc2C=C(c3c1cccc3)C4=CCN(CC4)C)C	CHEMBL94067	Doxepin
CN1CCC(=CC1)C2=Cc3ccccc3Oc4c2cccc4	CHEMBL163863	Doxepin
CN(C)CC1CC2c3ccccc3Sc4ccc(cc4C2O1)F	CHEMBL193639	Doxepin
CN(C)CC1CC2c3ccccc3Oc4ccc(cc4C2O1)F	CHEMBL194659	Doxepin
CN(C)CC1CC2c3ccccc3Oc4ccc(cc4C2O1)F	CHEMBL195706	Doxepin
CN1CCN(CC1)C2=Nc3cc(ccc3N(c4c2cccc4)N)Cl	CHEMBL215140	Doxepin
CN1CCN(CC1)C2=Cc3cc(ccc3Cc4c2cccc4)Cl	CHEMBL275696	Doxepin
CN(C)CCOC1=Cc2ccccc2Sc3c1cc(cc3)Cl	CHEMBL285802	Doxepin
CN(C)C1CCC2(C=C1)c3ccccc3C=Cc4c2cc(cc4)Cl	CHEMBL300647	Doxepin
CN1CCC(=CC1)C2=Cc3ccccc3C(=C)c4c2cccc4	CHEMBL327713	Doxepin
CN(C)CC1CC2c3ccccc3Sc4ccc(cc4C2O1)F	CHEMBL363581	Doxepin
CN(C)CC1CC2c3ccccc3Oc4ccc(cc4C2O1)Cl	CHEMBL364270	Doxepin
CN(C)CC1CC2c3ccccc3Sc4ccc(cc4C2O1)F	CHEMBL366164	Doxepin
CN1CCN(CC1)C2=Cn3cccc3Sc4c2cccc4	CHEMBL367045	Doxepin
CN(C)CC1CC2c3ccccc3Cc4ccccc4C2O1	CHEMBL371352	Doxepin
CN1CCN(CC1)C2=Nc3ccccc3Nc4c2cc(cc4)Cl	CHEMBL415300	Doxepin
CC1c2ccccc2C=C(c3c1cccc3)N4CCN(CC4)C	CHEMBL432762	Doxepin
CN(C)CC1CC2c3ccccc3Oc4ccc(cc4C2O1)Cl	CHEMBL435301	Doxepin
CN1CCC(CC1)C2=Cc3ccccc3Oc4c2cccc4	CHEMBL539057	Doxepin
CCN1CCC(=CC1)C2=Cc3ccccc3Cc4c2cccc4	CHEMBL542029	Doxepin
C=CCN1CCC(=CC1)C2=Cc3ccccc3Oc4c2cccc4	CHEMBL544418	Doxepin
CN1CCC(=CC1)C2=Cc3ccccc3Cc4c2cccc4	CHEMBL554670	Doxepin
C=CCN1CCC(=CC1)C2=Cc3ccccc3Cc4c2cccc4	CHEMBL555362	Doxepin
CCN1CCC(=CC1)C2=Cc3ccccc3Oc4c2cccc4	CHEMBL556969	Doxepin
CN1CCN(CC1)C2Cc3ccccc3Sc4c2cc(cc4)C#N	CHEMBL1259112	Doxepin
CN1CCN(CC1)C2Cc3ccccc3Sc4c2cc(cc4)CN	CHEMBL1259113	Doxepin
CN1CCc2c(c3cccc4c3n2-c5ccccc5CC4)C1	CHEMBL3104091	Doxepin
CCN1CCCC1CNC(=O)c2cc(ccc2OC)Br	CHEMBL1276268	Eticlopride
CCN1CCCC1CNC(=O)c2cccc(c2OC)O	CHEMBL1276269	Eticlopride

CCCc1ccc(c(c1)C(=O)NCC2CCCN2CC)OC	CHEMBL1276351	Eticlopride
CCc1ccc(c(c1)C(=O)NCC2CCCN2CC)OC	CHEMBL1276677	Eticlopride
CCc1cc(c(c(c1CC)C(=O)NCC2CCCN2CC)OC)O	CHEMBL1276647	Eticlopride
CCCc1cc(c(c(c1)O)OC)C(=O)NCC2CCCN2CC	CHEMBL1276585	Eticlopride
CCc1cc(c(c(c1)O)OC)C(=O)NCC2CCCN2CC	CHEMBL1276529	Eticlopride
CCN1CCCC1CNC(=O)c2c(c(cc(c2OC)O)C)C	CHEMBL1276528	Eticlopride
CCN1CCCC1CNC(=O)c2c(c(cc(c2OC)O)Cl)C	CHEMBL1276711	Eticlopride
CCN1CCCC1CNC(=O)c2c(c(cc(c2OC)Cl)O)OC	CHEMBL1276712	Eticlopride
CCCCc1ccc(c(c1OC)C(=O)NCC2CCCN2CC)OC	CHEMBL1276350	Eticlopride
CCN1CCCC1CNC(=O)c2c(ccc(c2Cl)Cl)OC	CHEMBL1276352	Eticlopride
CCc1ccc(c(c1OC)C(=O)NCC2CCCN2CC)OC	CHEMBL282925	Eticlopride
CCc1cc(c(c(c1Cl)C(=O)NCC2CCCN2CC)OC)O	CHEMBL1276560	Eticlopride
CCN1CCCC1CNC(=O)c2c(ccc(c2OC)O)OC	CHEMBL1276400	Eticlopride
CCN1CCCC1CNC(=O)c2cc(cc(c2OC)O)F	CHEMBL1276401	Eticlopride
CCCc1c(cc(c(c1C(=O)NCC2CCCN2CC)OC)O)C	CHEMBL1276766	Eticlopride
CCCc1cc(c(c(c1C)C(=O)NCC2CCCN2CC)OC)O	CHEMBL1276763	Eticlopride
CCN1CCCC1CNC(=O)c2c(ccc(c2OC)SC)OC	CHEMBL1276326	Eticlopride
CCN1CCCC1CNC(=O)c2cc(ccc2OC)Cl	CHEMBL1276238	Eticlopride
CCN1CCCC1CNC(=O)c2c(ccc(c2OC)O)Cl	CHEMBL1276399	Eticlopride
CCN1CCCC1CNC(=O)c2c(c(cc(c2Cl)C)O)OC	CHEMBL1276527	Eticlopride
COc1ccc(cc1C(=O)NCC2CCCN2CC=C)CCCF	CHEMBL428561	Eticlopride
CCc1cc(c(c(c1F)C(=O)NCC2CCCN2CC)OC)O	CHEMBL1276559	Eticlopride
CCN1CCCC1CNC(=O)c2cc(ccc2OC)S(=O)(=O)N	CHEMBL267044	Eticlopride
CCN1CCCC1CNC(=O)c2cc(ccc2OC)S(=O)(=O)N	CHEMBL26	Eticlopride
CCc1ccc(c(c1Cl)C(=O)NCC2CCCN2CC)OC	CHEMBL1276375	Eticlopride
CCc1c(cc(c(c1C(=O)NCC2CCCN2CC)OC)O)Cl	CHEMBL1276428	Eticlopride
CCN1CCCC1CNC(=O)c2cc(cc(c2OC)O)Cl	CHEMBL1276426	Eticlopride
CCN1CCCC1CNC(=O)c2c(cccc2OC)OC	CHEMBL282024	Eticlopride
CCN1CCCC1CNC(=O)c2c(ccc(c2OC)C)OC	CHEMBL1276645	Eticlopride
CCN1CCCC1CNC(=O)c2c(c(cc(c2OC)Cl)Cl)O	CHEMBL8809	Eticlopride
CCc1ccc(c(c1C(=O)NCC2CCCN2CC)OC)O	CHEMBL1276765	Eticlopride
CCN1CCCC1CNC(=O)c2cc(cc(c2OC)O)OC	CHEMBL1276586	Eticlopride
CCN1CCCC1CNC(=O)c2cc(cc(c2OC)O)C	CHEMBL1276742	Eticlopride
CCN1CCCC1CNC(=O)c2c(c(cc(c2Cl)OC)O)OC	CHEMBL1276709	Eticlopride
CCN1CCCC1CNC(=O)c2c(c(cc(c2Cl)Cl)O)OC	CHEMBL1276708	Eticlopride
CCc1cc(c(c(c1OC)C(=O)NCC2CCCN2CC)OC)O	CHEMBL1276327	Eticlopride
Clc1ccc(cc1)N1CCN(CCc2cc-3ccc[nH]c-3n2)CC1	CHEMBL347155	Piperidine/Piperazine
O=C1Cc2ccccc2N1CCCCN1CCN(CC1)c1ccccc1	CHEMBL395933	Piperidine/Piperazine
Cc1ccc(N2CCN(CC3CC3c3ccccc3)CC2)c(C)c1	CHEMBL2092961	Piperidine/Piperazine
COc1ccccc1N1CCN(CCc2ccc3[nH]nnc3c2)CC1	CHEMBL183776	Piperidine/Piperazine
CC1OC(=NC1CN1CCN(CC1)c1ccccc1)c1ccccc1	CHEMBL83306	Piperidine/Piperazine
CCON=C(CCN1CCN(CC1)c1nccs1)c1ccccc1	CHEMBL377388	Piperidine/Piperazine
O=c1[nH]c2ccccc2n1C1CCN(CCOc2ccccc2)CC1	CHEMBL2059304	Piperidine/Piperazine
C(N1CCN(CC1)c1cccnc1)c1c[nH]c(n1)-c1ccccc1	CHEMBL321513	Piperidine/Piperazine
O=C(NC1CCN(Cc2ccccc2)CC1)Nc1ccccn1	CHEMBL91272	Piperidine/Piperazine
OC1(CCN(Cc2c[nH]c3ccccc23)CC1)c1cccc(Cl)c1	CHEMBL396092	Piperidine/Piperazine
Fc1ccc(CCN2CCC3(CC2)CCc2ccccc2O3)c(F)c1	CHEMBL147431	Piperidine/Piperazine
Cc1cccc(N2CCN(CCCCNc3ccccn3)CC2)c1C	CHEMBL1223682	Piperidine/Piperazine
CCCN1CCC(CC1)c1noc2cc(F)ccc12	CHEMBL2207489	Piperidine/Piperazine
Fc1ccc(OCCCN2CCN(CC2)c2ccccn2)cc1	CHEMBL1940420	Piperidine/Piperazine
C(N1CCC(CC1)n1cccc1-c1cnco1)c1ccccc1	CHEMBL104453	Piperidine/Piperazine
COc1ccccc1N1CCN(Cc2ccc(OCCF)cc2)CC1	CHEMBL271513	Piperidine/Piperazine
C(N1CCC(CC1)n1cc(nn1)-c1ccccc1)c1ccccc1	CHEMBL24170	Piperidine/Piperazine

Clc1cccc(N2CCN(Cc3cccnc3)CC2)c1Cl	CHEMBL2420780	Piperidine/Piperazine
OC(COc1ccccc1)CN1CCC(CC1)Oc1ccccc1	CHEMBL32402	Piperidine/Piperazine
C(Oc1ccccc1)C1CCC2CN(CCN2C1)c1ncccn1	CHEMBL162265	Piperidine/Piperazine
COc1ccccc1C1CCN(CNC(=O)c2cccc(C)c2)CC1	CHEMBL376092	Piperidine/Piperazine
C(CN1CCC(COCC=Cc2ccccc2)CC1)Cc1ccccc1	CHEMBL308980	Piperidine/Piperazine
Cn1c(CN2CCC(CC2)c2ccccc2)nc2ccccc12	CHEMBL593196	Piperidine/Piperazine
C(CN1CCN(CC1)c1ccccc1)Cc1ccc2nc[nH]c2c1	CHEMBL2298813	Piperidine/Piperazine
Cc1ccc(cc1)C1CCN(Cc2ccc3OCC(=O)Nc3c2)CC1	CHEMBL346398	Piperidine/Piperazine
COc1ccc(cc1)N1CCN(CCc2c[nH]c3ncccc23)CC1	CHEMBL347291	Piperidine/Piperazine
Cc1c(nc(O)n1C1CCN(Cc2ccccc2)CC1)-c1ccccn1	CHEMBL91553	Piperidine/Piperazine
O=C1N(CCCCN2CCN(CC2)c2ccccc2)Cc2ccccc12	CHEMBL240774	Piperidine/Piperazine
CCCCN1CCN(CC1)c1ccccc1OC	CHEMBL26789	Piperidine/Piperazine
CCCN1CCN(CC1)c1cccc(c1)S(C)(=O)=O	CHEMBL596801	Piperidine/Piperazine
CS(=O)(=O)c1cccc(c1)C1CCN(Cc2ccccc2)CC1	CHEMBL596800	Piperidine/Piperazine
CON=C(CCN1CCN(CC1)c1ccccn1)c1cccc(C)c1	CHEMBL212903	Piperidine/Piperazine
CCOc1ccccc1N1CCN(Cc2cccc3ccccc23)CC1	CHEMBL2420781	Piperidine/Piperazine
CCCCC1CCN(CCCC(=O)c2ccccc2C)CC1	CHEMBL1242950	Piperidine/Piperazine
Fc1ccc(CCCCN2CCN(CC2)c2ccc(Cl)cc2)cc1	CHEMBL1940408	Piperidine/Piperazine
CNc1nc(Cl)c(Sc2ccccc2)c(n1)N1CCN(C)CC1	CHEMBL14899	Piperidine/Piperazine
C(N1CCN(CC1)c1ccccc1)c1cnn(c1)-c1ccccc1	CHEMBL210405	Piperidine/Piperazine
C(N1CCN(CC1)c1cccc2OCCOc12)c1c[nH]c2ccccc12	CHEMBL195015	Piperidine/Piperazine
C(N1CCN(CC1)c1ncccn1)c1cccc(c1)-c1ccsc1	CHEMBL64597	Piperidine/Piperazine
Cc1cc2CCN3CCC(O)(CC3c2cc1O)c1ccc(Cl)cc1	CHEMBL2331599	Piperidine/Piperazine
CCCN1CCC(CC1)c1cn(C)c2ccc(F)cc12	CHEMBL2207486	Piperidine/Piperazine
Fc1ccc(CCCCN2CCN(CC2)c2ncccn2)cc1	CHEMBL1940415	Piperidine/Piperazine
CON=C(CCN1CCN(CC1)c1ccccn1)c1cccc(F)c1	CHEMBL213164	Piperidine/Piperazine
C(Cc1ccccc1)N1CCC(CC1)c1cc([nH]n1)-c1cccs1	CHEMBL77743	Piperidine/Piperazine
C(Cc1ccccc1)N1CCC(CC1)c1cc(n[nH]1)C1CCCCC1	CHEMBL77744	Piperidine/Piperazine
COc1ccccc1N1CCN(CNC(=O)c2ccc(C)cc2)CC1	CHEMBL51023	Piperidine/Piperazine
N#CC(=Cc1cc(CN2CCN(CC2)c2ccccc2)c[nH]1)C#N	CHEMBL294459	Piperidine/Piperazine
C(N1CCN(CC1)c1cccc2[nH]ccc12)c1ccccc1	CHEMBL328051	Piperidine/Piperazine
