# Supplementary material for: Performance of virtual screening against GPCR homology models: Impact of template selection and treatment of binding site plasticity
Source: PLoS Comput Biol. 2020 Mar 13;16(3):e1007680. doi: 10.1371/journal.pcbi.1007680 (PMC7135368; doi:10.1371/journal.pcbi.1007680)
Supplement: S9 Table — (PDF) [file pcbi.1007680.s009.pdf]

S9 Table. Average RMSDs to the starting structure (homology model) of MD snapshots of the D_2R and 5-$HT_{2A}R$.

State (template)[a]	MD Trajectory[b]	RMSD (Å)[c] TMBB	BSBB	Ligand
		**D_2R**		
**Apo (D_3R)**	1	1.8 ± 0.3	1.9 ± 0.4	-
	2	1.9 ± 0.2	1.9 ± 0.3	-
	3	1.6 ± 0.1	1.6 ± 0.2	-
**Apo (Rho)**	1	2.4 ± 0.2	2.4 ± 0.1	-
	2	2.3 ± 0.1	2.4 ± 0.2	-
	3	2.4 ± 0.2	2.3 ± 0.2	-
**Holo (D_3R)**	1	1.3 ± 0.1	1.2 ± 0.2	1.2 ± 0.3
	2	1.6 ± 0.2	1.2 ± 0.2	1.0 ± 0.2
	3	1.4 ± 0.1	1.6 ± 0.3	1.2 ± 0.2
		**5-$HT_{2A}R$**		
**Apo (5-$HT_{2C}R$)**	1	1.7 ± 0.2	1.4 ± 0.1	-
	2	2.2 ± 0.3	1.6 ± 0.2	-
	3	1.7 ± 0.2	1.3 ± 0.1	-
**Apo (Rho)**	1	3.4 ± 0.4	3.8 ± 0.4	-
	2	3.4 ± 0.4	3.9 ± 0.4	-
	3	3.2 ± 0.2	3.6 ± 0.2	-
**Holo (5-$HT_{2C}R$)**	1	2.2 ± 0.3	1.4 ± 0.2	1.1 ± 0.2
	2	2.1 ± 0.2	1.4 ± 0.2	1.0 ± 0.2
	3	2.0 ± 0.3	1.3 ± 0.1	1.0 ± 0.2

[a] MD simulations were carried out in the absence (apo) and presence (holo) of orthosteric ligand. The template used to create the homology model is shown in parenthesis.
[b] Three MD simulation trajectories of 100 ns were carried out for each homology model.
[c] RMSD ± standard deviation (1000 snapshots from 100 ns) for the TM backbone (TMBB), BS backbone (BSBB), and orthosteric ligand.
