# Supplementary material for: Performance of virtual screening against GPCR homology models: Impact of template selection and treatment of binding site plasticity
Source: PLoS Comput Biol. 2020 Mar 13;16(3):e1007680. doi: 10.1371/journal.pcbi.1007680 (PMC7135368; doi:10.1371/journal.pcbi.1007680)
Supplement: S10 Table — Statistics are based on 50 snapshots per trajectory. (PDF) [file pcbi.1007680.s010.pdf]

S10 Table. Ligand enrichments (aLogAUC) for MD simulation snapshots of the D_2R and 5-$HT_{2A}R$ homology models. Statistics are based on 50 snapshots per trajectory.

State (template)[a]	MD Trajectory[b]	aLogAUC		
		Median	Max	Ensemble[c]
		D_2R		
Apo (D_3R)	1	12.7	24.5	16.9
	2	14.1	20.4	17.5
	3	8.8	17.1	16.2
Holo (D_3R)	1	11.9	19.1	16.9
	2	11.7	21.3	18.2
	3	10.1	16.2	16.4
		5-$HT_{2A}R$		
Apo (5-$HT_{2C}R$)	1	10.5	22.5	16.3
	2	10.5	18.5	10.0
	3	11.6	21.4	13.0
Holo (5-$HT_{2C}R$)	1	24.2	29.1	26.1
	2	25.0	29.8	27.7
	3	25.0	30.9	27.8

[a]MD simulations were carried out in the absence (apo) and presence (holo) of orthosteric ligand. The template used to create the homology model is shown in parenthesis.
[b]Three MD simulations of 100 ns were carried out for each homology model and 50 snapshots from clustering of each trajectory were analyzed.
[c]The ensemble aLogAUC was calculated by identifying the best docking score of each docked compound among multiple MD snapshots, leading to a single aLogAUC value for the set. The ensemble enrichment was calculated for 50 snapshots from clustering.
