# Supplementary figures and images for: Performance of virtual screening against GPCR homology models: Impact of template selection and treatment of binding site plasticity
Source: PLoS Comput Biol. 2020 Mar 13;16(3):e1007680. doi: 10.1371/journal.pcbi.1007680 (PMC7135368; doi:10.1371/journal.pcbi.1007680)

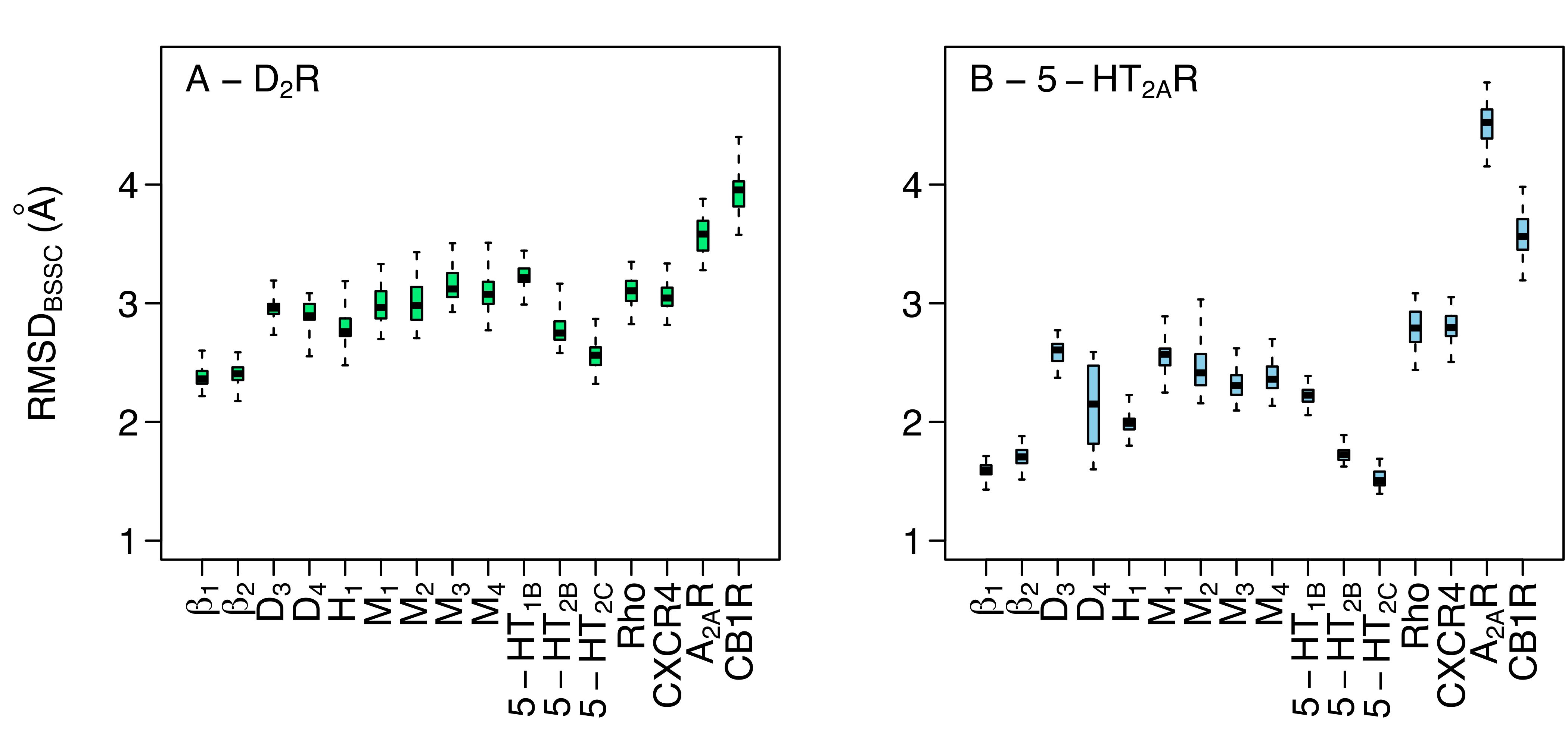

Supplement: S1 Fig — Distributions of the RMSDBSSC to the crystal structures for 50 models of the D2R (A) and 5-HT2AR (B) based on different templates using a boxplot representation. The box represents the 50th percentile of the data and the black band shows the median value. The lowest and highest RMSDBSSC values are represented by the whiskers. (TIF) [file pcbi.1007680.s011.tif]

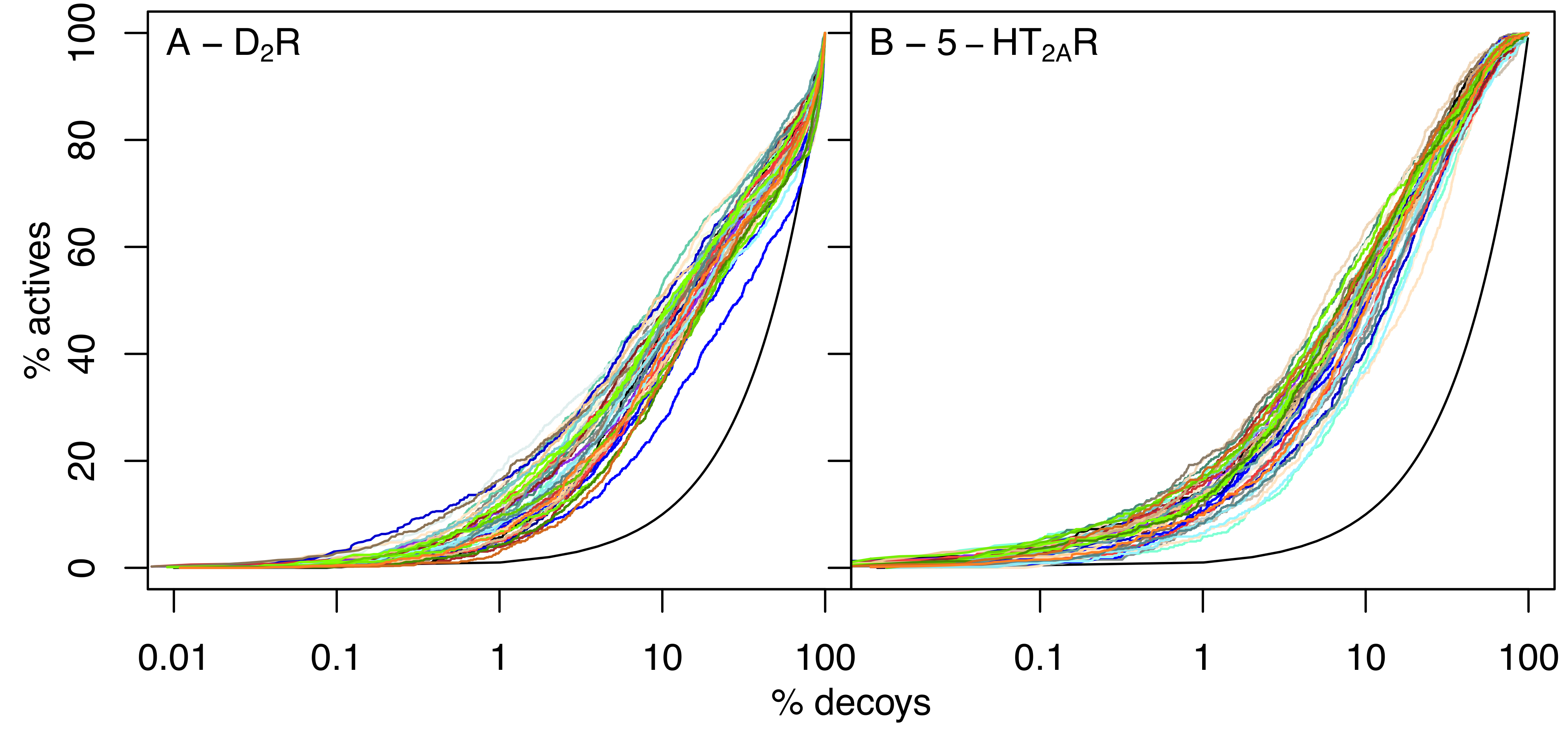

Supplement: S2 Fig — Enrichment curves for 50 (A) D2R (based on D3R template) and (B) 5-HT2AR (based on 5-HT2CR template) homology models. Receiver operating characteristic (ROC) curves for databases of ligands and property-matched decoys ranked by molecular docking. The percentage of ligands identified and decoys found are shown on the y- and x-axis, respectively. The solid black line represents random enrichment of ligands. (TIF) [file pcbi.1007680.s012.tif]

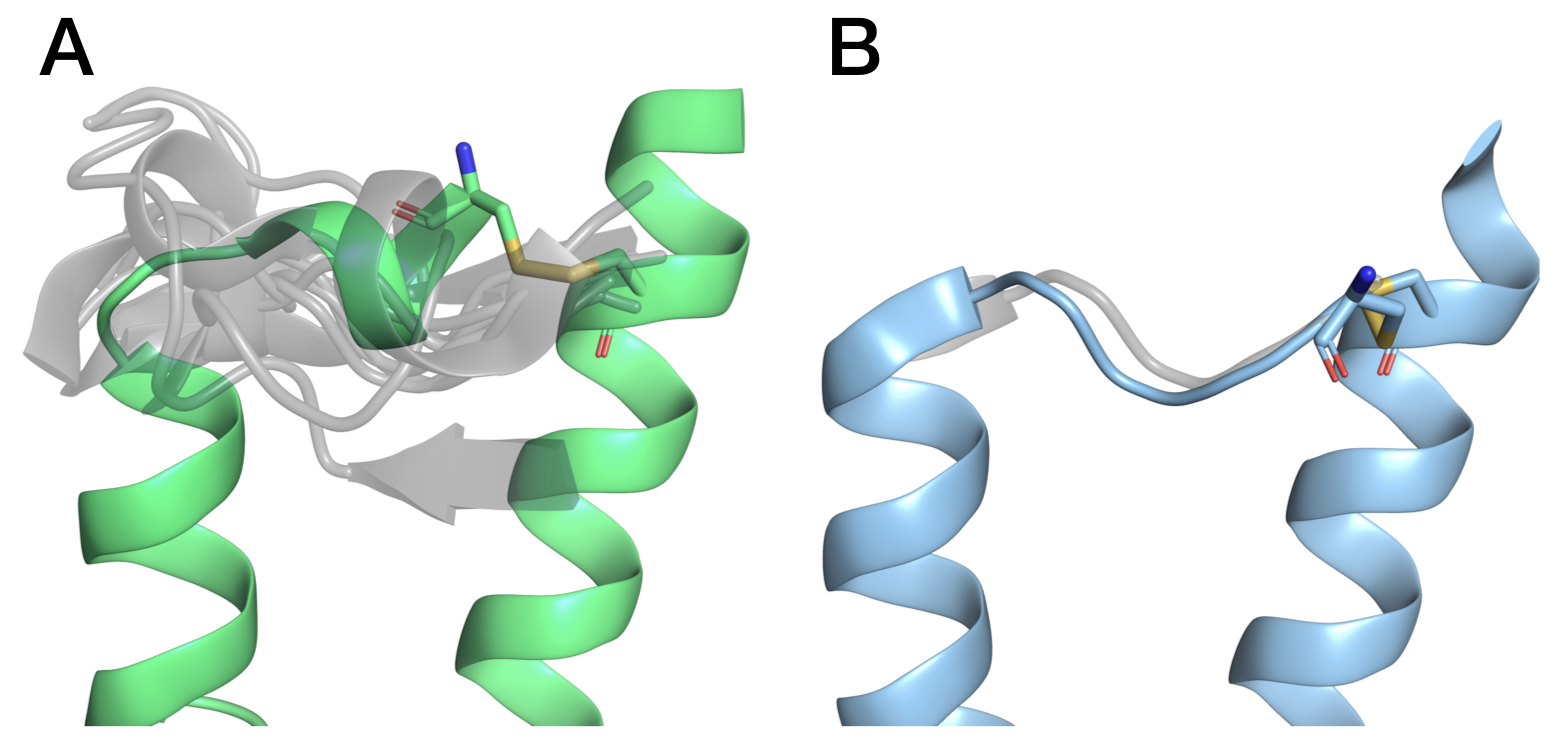

Supplement: S3 Fig — (A) Comparison of ECL2 of the D2R (green) to templates (β1AR, D3R, D3R, H1R, M2R, 5-HT1BR, 5-HT2BR, 5-HT2CR, A2AAR, CB1R, CXCR4, Rho; grey). (B) Comparison of the ECL2 of the 5-HT2AR (blue) to the 5-HT2CR template (grey). The receptor backbone is shown as cartoons. The conserved cysteine bridge formed by Cys45.50 is shown as sticks. (TIF) [file pcbi.1007680.s013.tif]

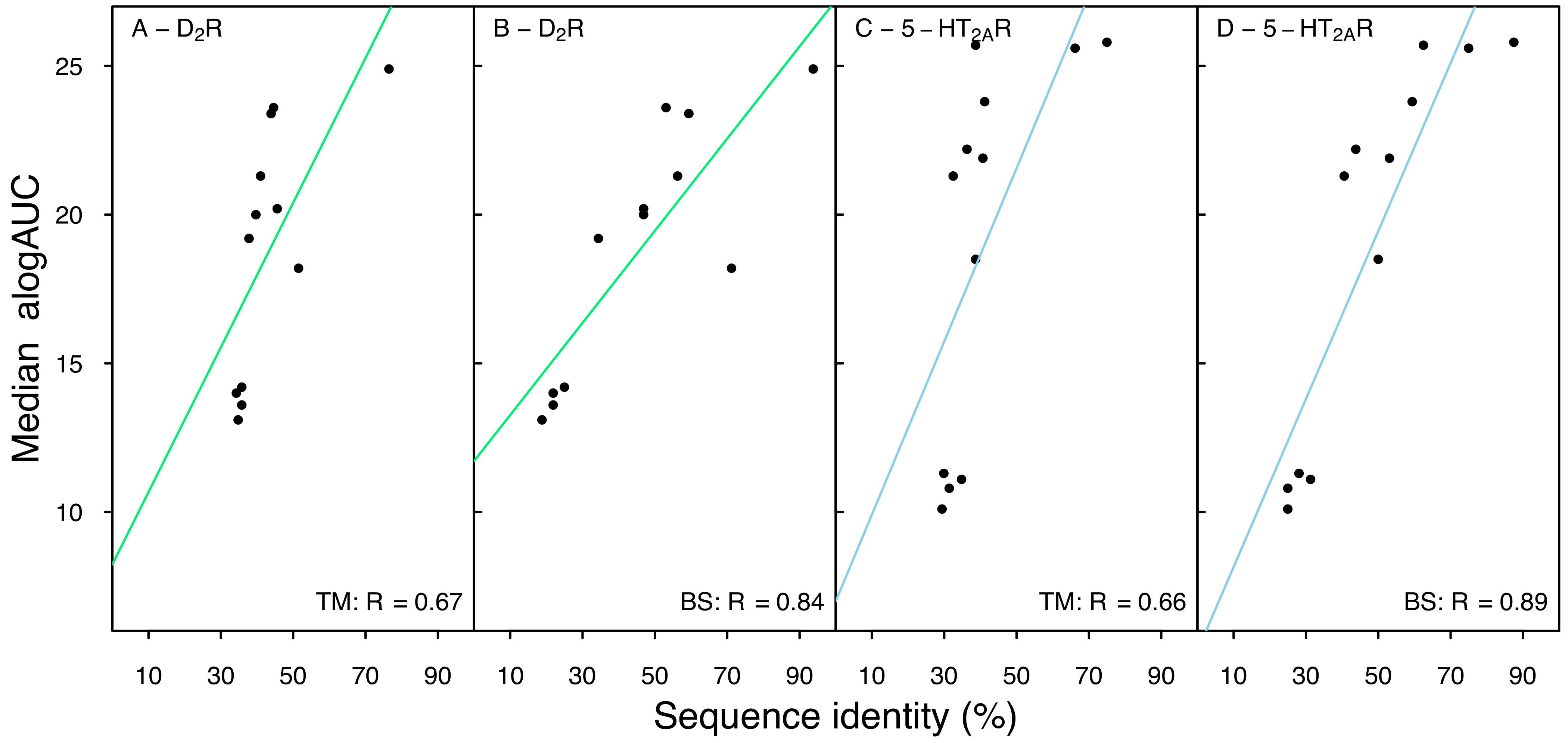

Supplement: S4 Fig — The median aLogAUC values of the D2R (A-B) and 5-HT2AR (C-D) homology models without ECL2 based on aminergic templates with different TM (A and C) or BS (B and D) sequence identities. The solid line represents a linear regression and R is Pearson’s correlation coefficient. (TIF) [file pcbi.1007680.s014.tif]

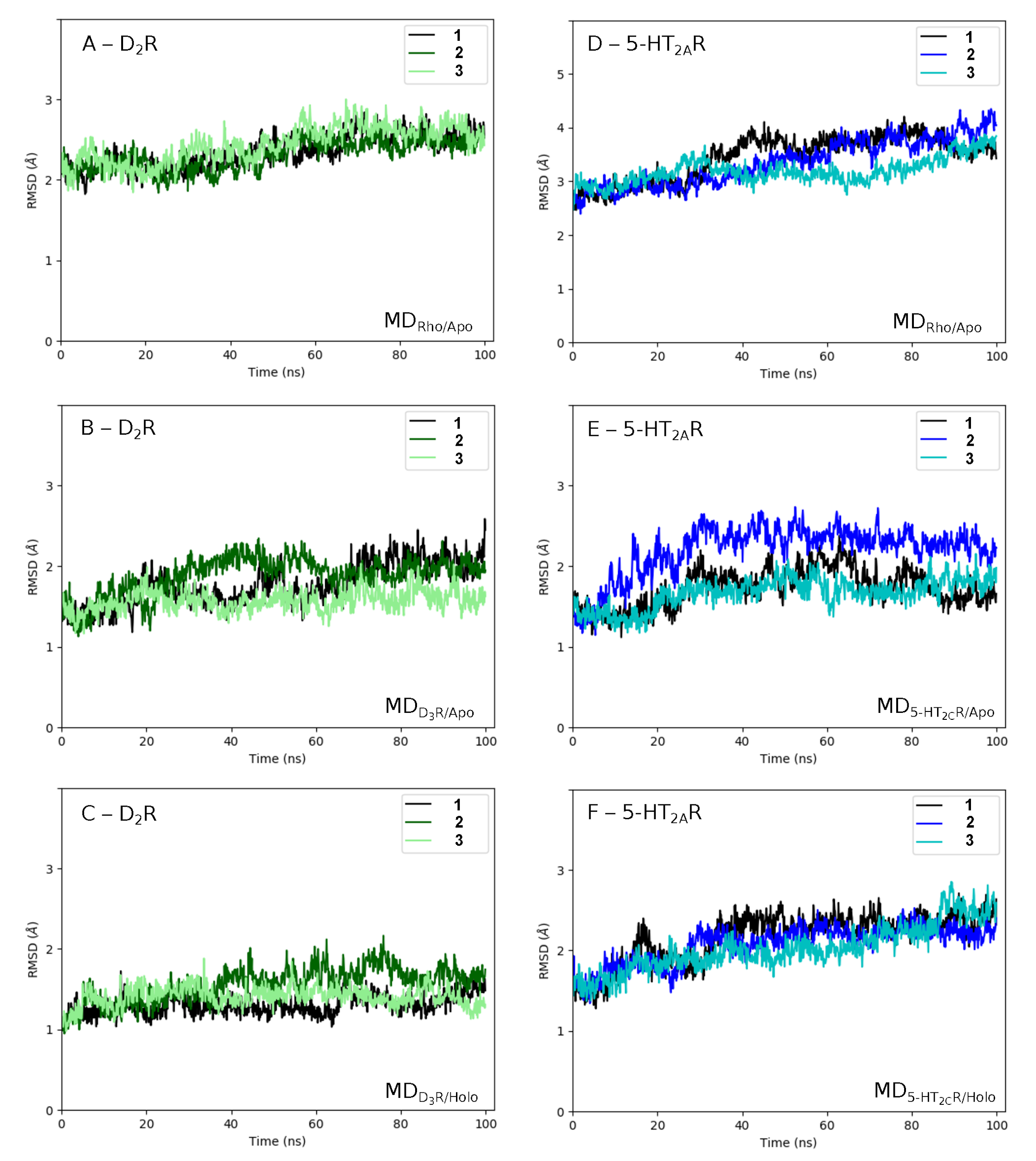

Supplement: S5 Fig — TM backbone RMSDs of the D2R (A-C) and 5-HT2AR (D-F) MD snapshots to the initial homology model. The three trajectories of the Rho-based models (MDRho/Apo, A and D) and models based on the most closely related template in apo (MDTemplate/Apo, B and E) and holo forms (MDTemplate/Holo, C and F) are shown. (TIF) [file pcbi.1007680.s015.tif]
